# CTCF is selectively required for maintaining chromatin accessibility and gene expression in human erythropoiesis

**DOI:** 10.1186/s13059-025-03510-z

**Published:** 2025-02-28

**Authors:** Xue Yang, Li Cheng, Ye Xin, Jianxiang Zhang, Xinfeng Chen, Jinchao Xu, Mengli Zhang, Ruopeng Feng, Judith Hyle, Wenjie Qi, Wojciech Rosikiewicz, Beisi Xu, Chunliang Li, Peng Xu

**Affiliations:** 1 Cyrus Tang Medical Institute, National Clinical Research Center for Hematologic Diseases, State Key Laboratory of Radiation Medicine and Protection, Collaborative Innovation Center of Hematology, Suzhou Medical College, Soochow University, Suzhou, Jiangsu Province 215123 China; 2 Department of Hematology, St. Jude Children’s Research Hospital, Memphis, TN 38105 USA; 3 Department of Tumor Cell Biology, St. Jude Children’s Research Hospital, Memphis, TN 38105 USA; 4 Center for Applied Bioinformatics, St. Jude Children’s Research Hospital, Memphis, TN 38105 USA; 5 Present address: GenAssist Therapeutics Incorporation, Suzhou, Jiangsu Province 215000 China

**Keywords:** CTCF, Chromatin accessibility, Transcription regulation, Hematopoiesis, Erythropoiesis, Genome editing

## Abstract

**Background:**

CTCF is considered as the most essential transcription factor regulating chromatin architecture and gene expression. However, genome-wide impact of CTCF on erythropoiesis has not been extensively investigated.

**Results:**

Using a state-of-the-art human erythroid progenitor cell model (HUDEP-2 and HEL cell lines), we systematically investigate the effects of acute CTCF loss by an auxin-inducible degron system on transcriptional programs, chromatin accessibility, CTCF genome occupancy, and genome architecture. By integrating multi-omics datasets, we reveal that acute CTCF loss notably disrupts genome-wide chromatin accessibility and the transcription network. We detect over thousands of decreased chromatin accessibility regions but only a few hundred increased regions after CTCF depletion in HUDEP-2 and HEL lines, suggesting the role of CTCF in maintaining proper chromatin openness in the erythroid lineage. CTCF depletion in the erythroid context notably disrupts the boundary integrity of topologically associating domains and chromatin loops but does not affect nuclear compartmentalization. We find erythroid lineage-specific genes, including some metabolism-related genes, are suppressed at immature and mature stages. Notably, we find a subset of genes whose transcriptional levels increase upon CTCF depletion, accompanied by decreased chromatin accessibility regions enriched with the GATA motif. We further decipher the molecular mechanism underlying the CTCF/GATA2 repression axis through distal non-coding chromatin regions. These results suggest a suppressive role of CTCF in gene expression during erythroid lineage specification.

**Conclusions:**

Our study reveals a novel role of CTCF in regulating erythroid differentiation by maintaining its proper chromatin openness and gene expression network, which extends our understanding of CTCF biology.

**Supplementary Information:**

The online version contains supplementary material available at 10.1186/s13059-025-03510-z.

## Background

Erythroid or red blood cells are generated from hematopoietic stem and progenitor cells through a stepwise differentiation process known as erythropoiesis. Erythropoiesis provides a valuable model for understanding metazoan gene regulation, as erythroblast progenitors undergo several specialized cell divisions associated with multiple regulatory events, including global gene transcriptome and proteome remodeling, global DNA methylation, and nuclear chromatin architecture remodeling [1–5]. Although more than half of topologically associating domains (TADs) are disrupted from proerythroblasts to late orthochromatic erythroblasts during the process of chromatin condensation in both mice and human erythropoiesis [6–8], the mechanisms underlying chromatin structure remodeling during this process remain largely unknown. The CCCTC binding factor (CTCF) is the best-known master regulator of mammalian three-dimensional (3D) genome architecture maintenance and gene expression regulation [9]. CTCF is required for long-term hematopoietic stem cell (HSC) proliferation and differentiation as it governs the expression of multiple stemness genes [10]. Moreover, CTCF functions in the differentiation of multiple hematopoietic lineages, including the lymphoid T, B, and myeloid cells [11–13]. More recent work revealed that the promoter-proximal CTCF binding site on *Runx1* plays a key role in establishing its chromatin architecture in hematopoiesis [14]. Nevertheless, the suppressive role of CTCF in gene transcription and how CTCF coordinates with other TFs in lineage differentiation are not fully understood.

In this study, we established the auxin-inducible degradation (AID) system as CTCF-AID knockin in two human erythroid cell lines, HUDEP-2 and HEL, which are well-known cellular models for studying erythroid gene expression and differentiation [15, 16]*.* We comprehensively profiled and analyzed the transcriptome RNA sequencing (RNA-seq), transposase-accessible chromatin using sequencing (ATAC-seq), chromatin immunoprecipitation sequencing (ChIP-seq) for CTCF and GATA1, and the in situ high-throughput chromosome conformation capture (Hi-C) for 3D genome architecture in the presence or absence of CTCF by combining publicly available datasets. Strikingly, thousands of chromatin accessibility regions were decreased after the depletion of the CTCF protein in the HUDEP-2 and HEL lines. In contrast, only a few hundred increased regions were observed. We further revealed a stage-dependent role of CTCF in gene regulation during erythroid maturation and identified that the expression of erythroid-related genes, including genes involved in amino acid and heme metabolism, was suppressed in the immature and mature stages. Moreover, our data confirmed that acute depletion of CTCF significantly disrupted the TAD boundary integrity and chromatin loops but did not affect nuclear compartmentalization. Notably, the loss of chromatin looping after CTCF depletion significantly correlated with decreased chromatin accessibility and decreased gene transcription. We further functionally validated the selective gene regulation patterns via genome editing and combinatorial multi-omics profiling. Therefore, we propose that CTCF plays a critical role in maintaining chromatin accessibility and gene expression in human erythropoiesis.

## Results

### CTCF is indispensable for the proliferation and maturation of erythroid progenitor cells

**Fig. 1:**
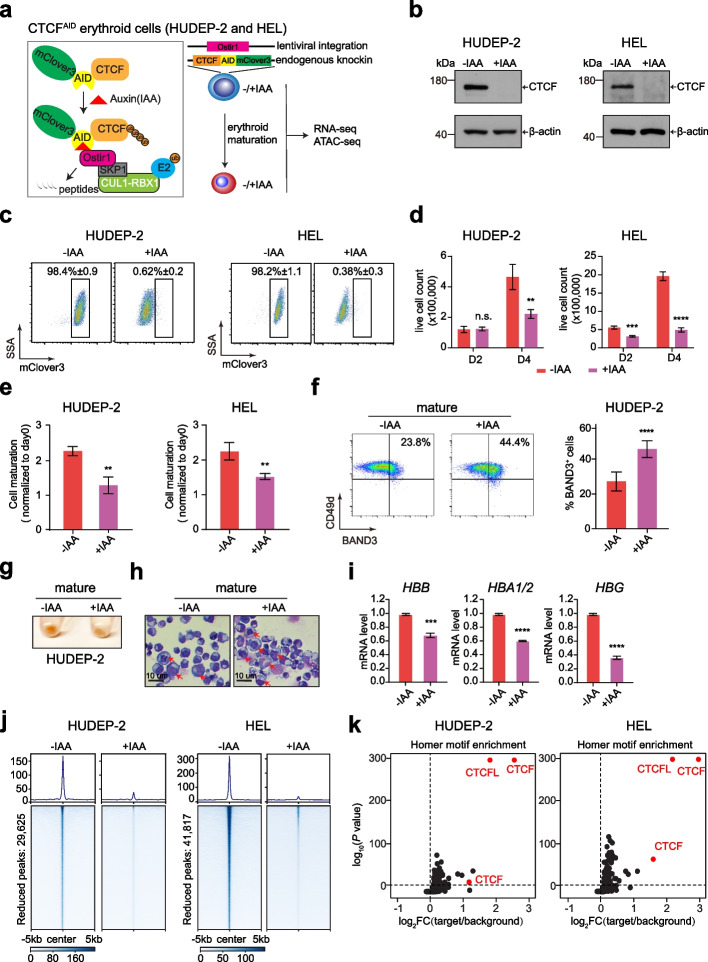
CTCF is indispensable for the proliferation and maturation of erythroid progenitor cells. **a** Schematic diagram of the auxin-inducible degron model for tagging endogenous CTCF in HUDEP-2 and HEL cells. The miniAID-mClover3 cassette was electroporated to knockin both endogenous alleles in frame with CTCF, and a lentivirus expressing plant OsTIR1 was transduced into HUDEP-2 and HEL cells (right panel). As shown in the left panel, in the presence of auxin (IAA), the plant E3 ligase adaptor OsTIR1 combines with Skip1/Cullin scaffold components to form the functional SCF-OsTIR1 E3 ubiquitin ligase complex, which rapidly ubiquitinates and degrades the miniAID-mClover3 fusion protein. **b** Western plots showing CTCF expression in HUDEP-2 and HEL cell lines without IAA treatment and with IAA treatment for 24 h in an expansion medium. CTCF was detected with anti-CTCF antibodies. β-actin was used as a loading control. **c** Flow cytometry plots showing CTCF-AID-mClover3 expression without and with IAA treatment for 24 h in the HUDEP-2 and HEL cell lines. The data shown are the means ± SEMs from three independent experiments. **d** Proliferation of HUDEP-2 and HEL cells on day 2 and day 4 after induction of erythroid maturation without and with IAA treatment for 24 h. The data shown are the means ± SEMs from three independent experiments. The “n.s.” represents nonsignificant; ** *P* < 0.01, *** *P* < 0.001, **** *P* < 0.0001, unpaired Student’s *t* test. **e** HUDEP-2 and HEL cell maturation after induction of CTCF-AID degradation with 500 µM IAA for 24 h. The data are presented as the means ± SEMs from three independent experiments. ** *P* < 0.01, unpaired Student’s *t* test. **f** Flow cytometry plots showing BAND3 and CD49d expression in HUDEP-2 cells on day 2 after induction of erythroid maturation without and with IAA treatment for 24 h. The graph on the right shows the quantified BAND3^+^ cell percentage. The data are presented as the means ± SEMs from three independent experiments. **** *P* < 0.0001, unpaired Student’s *t* test. **g** Images of HUDEP-2 cell pellets after 2 days of induced erythroid maturation showing hemoglobinization without and with IAA treatment for 24 h. **h** May-Grünwald-Giemsa-stained HUEDP-2 cells after erythroid maturation on day 4 without and with IAA treatment for 24 h. Red arrows denote immature erythroblasts. Scale bar: 10 µm. **i** *HBB*, *HBA1/2*, and *HBG* mRNA levels in HUDEP-2 erythroid maturation on day 4 without and with IAA treatment. The *y*-axis shows the mRNA level relative to β-actin. ** *P* < 0.01, *** *P* < 0.001, **** *P* < 0.0001, unpaired Student’s *t* test. **j** Genomic heatmap centered at reproducible CTCF-reduced peaks summits by CTCF ChIP-seq data from CTCF-AID cells before and after IAA treatment for 24 h (29,625 peaks in HUDEP-2 cells and 41,817 in HEL cells). **k** Homer motif enrichment analysis revealed that the CTCF and CTCFL motifs were the top enriched transcription factor motifs for the CTCF-reduced regions in both HUDEP-2 and HEL cells. The enriched CTCF motifs are shown as red circles

To identify the immediate role of CTCF in erythroid cell genome features and related erythroid gene expression, we utilized the previously established CTCF-AID knockin HUDEP-2 cell model [17] by integrating the miniAID-mClover3 cassette into the endogenous *CTCF* locus, accompanied by stable expression of the plant E3 ubiquitin ligase Ostir1 (Fig. 1a). To validate the major conclusion in an alternative model, we also successfully established a new CTCF-AID system in erythroleukemia cell line, HEL. In the presence of the plant hormone auxin or indole-3-acetic acid (IAA), OsTIR1 can quickly mediate the ubiquitination of the CTCF fusion protein and further degradation by the proteasome system. We confirmed that the CTCF protein was efficiently depleted after treatment with IAA for 24 h, accompanied by a reduction in the Clover3 fluorescence signal in both CTCF-AID cell lines (Fig. 1b and c; Additional file 1: Fig. S1a). The CTCF mRNA level was slightly increased upon IAA treatment (Additional file 1: Fig. S1b), suggesting a feedback or compensatory effect after CTCF protein degradation. Moreover, the protein degradation was reversible after removing the IAA from the culture medium (Additional file 1: Fig. S1a).

Next, we studied the effects of erythroid progenitor cell proliferation and maturation. Consistent with previous findings that CTCF functions as an essential gene, we observed that the loss of CTCF protein over 48 and 72 h significantly blocked the cell growth of both HUDEP-2 and HEL cells. However, no severe impact on cell growth was observed within 24 h (Fig. 1d; Additional file 1: Fig. S1c). Consistently, acute CTCF loss did not result in significant apoptosis in either HUDEP-2 or HEL cells within 24 h of IAA treatment (Additional file 1: Fig. S1d). Therefore, we reasoned that utilizing the 24 h IAA treatment regimen would be optimal for subsequent mechanistic studies to reduce secondary effects.

The HUDEP-2 and HEL cell lines can be induced to undergo terminal maturation after treatment with EPO and hemin, respectively. Although the cells treated with IAA exhibited no difference in expansion conditions within 24 h, the loss of CTCF inhibited cell growth during erythroid maturation (Fig. 1e). Moreover, the CTCF depletion also increased the level of the late erythroid maturation cell surface marker BAND3, as suggested by the increase in BAND3-positive erythroid cell number from 25 to 45% in HUDEP-2 line, which was accompanied by less hemoglobinization and larger cell size (Fig. 1f–h). Similarly, we observed that CTCF depletion in HEL cell line decreased the CD71 expression after hemin-induced maturation (Additional file 1: Fig. S1e). To further explore the effect of CTCF loss on globin expression, we measured both adult and fetal globin expressions with and without CTCF. We found that CTCF is required for the programmed expression of both adult and fetal globin genes in these two cell lines (Fig. 1i, Additional file 1: Fig. S1f). These results suggest that CTCF is required for normal erythroid proliferation and maturation in erythroid progenitors.

To examine the effects of CTCF depletion on the occupancy of CTCF at genomic loci, we performed ChIP-seq to identify the CTCF binding profile in CTCF-AID HUDEP-2 and HEL cells before and after IAA treatment for 24 h. Principal component analysis (PCA) confirmed that before and after IAA treatment groups were separated from each other, suggesting a global dynamic change in CTCF occupancy (Additional file 1: Fig. S1g). Indeed, a global reduction of CTCF occupancy was observed in both HUDEP-2 and HEL cells after the 24 h of IAA treatment, with a proportion of retained regions (Fig. 1j; Additional file 1: Fig. S1h). Moreover, the Homer motif analysis revealed that the CTCF consensus binding motif was the top enriched TF motif for the CTCF-reduced regions (Fig. 1k). Collectively, these results confirmed the efficient elimination of chromatin-bound CTCF in the erythroid progenitor cells.

### CTCF depletion leads to substantial loss of chromatin accessibility in erythroid progenitor cells

**Fig. 2:**
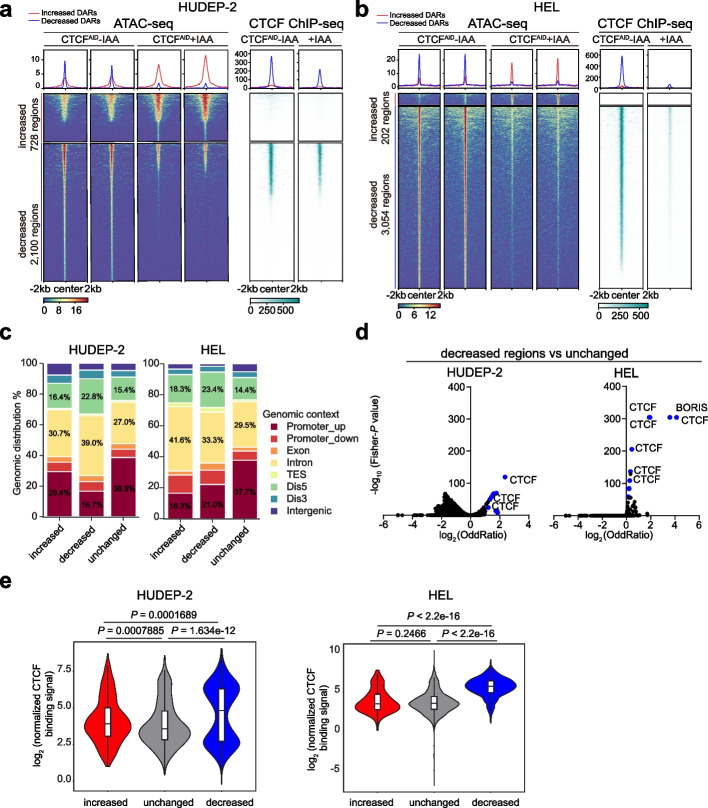
CTCF depletion leads to substantial loss of chromatin accessibility in erythroid progenitor cells. **a** Heatmap centered at ATAC-seq nucleosome-free peak summits for 728 increased regions and 2100 decreased regions together with the corresponding CTCF binding signal by the ChIP-seq in the CTCF-AID HUDEP-2 cell line without and with IAA treatment for 24 h. CTCF ChIP-seq data indicate that decreased DARs have the most robust CTCF binding in HUDEP-2 cells. Increased and decreased regions were called based on a cutoff of false discovery rate (FDR) < 0.05 and fold change > 2. **b** Heatmap centered at ATAC-seq nucleosome-free peak summits for 202 increased regions and 3054 decreased regions together with the corresponding CTCF binding signal determined by ChIP-seq in the CTCF-AID HEL cell line without and with IAA treatment for 24 h. CTCF ChIP-seq data indicate decreased DARs have the most robust CTCF binding in HEL cells. Increased and decreased regions were called based on a cutoff of false discovery rate (FDR) < 0.05 and fold change > 2. **c** Genomic localization of increased, decreased, and unchanged regions from ATAC-seq in the HUDEP-2 and HEL cell lines without and with IAA treatment for 24 h. The peak distributions of increased, decreased, and control regions from ATAC-seq in the HUDEP-2 and HEL cell lines were calculated based on the absolute distance to the TSS. **d** Volcano plot of motif enrichment analysis of ATAC-seq comparisons: decreased regions versus unchanged control regions in the HUDEP-2 and HEL cell lines. The *P* values and odds ratios were calculated via Fisher’s exact tests to compare the frequency of regions containing a motif with those that do not. Each dot represents a motif in the database. The blue dots indicate motifs enriched for decreased regions. For the highlighted CTCF, multiple dots are shown as different motif IDs enriched in the database. **e** Violin plot of the normalized CTCF binding signal (fragments per kilobase of peaks per million reads mapped, FPKM) at decreased DARs and increased DARs or control regions in the CTCF-AID HUDEP-2 (left panel) and HEL (right panel) cell lines, from which the relative CTCF binding signal was calculated. **** *P* < 0.0001 according to the Wilcoxon test

Given that CTCF directly binds to DNA with consensus motifs and that the immediate loss of CTCF may change the local chromatin environment, we hypothesized that CTCF maintains appropriate chromatin accessibility in erythroid progenitors. To test this hypothesis, a genome-wide ATAC-seq was performed in both HUDEP-2 and HEL cells before and after the 24 h of IAA treatment. A total of 51,004 and 71,039 reproducible peaks were identified from two respective cell lines. The differential accessibility regions (DARs) were defined by a stringent cutoff (false discovery rate (FDR) controlled *P*-value < 0.05 and fold change > 2) [18]. We observed that the decreased DARs were notably more than the increased regions after the CTCF depletion in both erythroid cell lines. After acute depletion of CTCF in the HUDEP-2 cell line, about 2100 chromatin accessibility regions were significantly decreased, whereas only 728 regions were significantly increased (Fig. 2a; Additional file 1: Fig. S2a; Additional file 2: Table S1). Similarly, 3054 chromatin accessibility regions were decreased, whereas only 202 regions were increased in the HEL line (Fig. 2b; Additional file 1: Fig. S2a; Additional file 3: Table S2).

Given the unique feature of substantially decreased regions in erythroid progenitors, we analyzed their genomic distribution and calculated the genomic distance from the chromatin accessibility regions to the nearest CTCF motifs. We observed that more than half of the decreased regions were located at enhancers, either distal regulatory regions (defined by 50 kb > absolute distance to the transcription start site > 2 kb) or introns. In contrast, the increased or unchanged regions were closer to the gene promoter regions (Fig. 2c). The decreased regions were closer to the CTCF motifs than the increased or unchanged regions (Additional file 1: Fig. S2b). Next, we predicted the TF occupancy profiles of DARs using the TRANSFAC motif database as a reference [19] and scored the enrichment frequency. The top TFs enriched for the decreased regions were CTCF and CTCFL (Fig. 2d; Additional file 4: Table S3). In contrast, several master TFs involved in regulating erythropoiesis and hematopoiesis (e.g., GATA1/2, TAL1, MEIS1, and NFY) were significantly enriched in increased regions (Additional file 4: Table S3). To further confirm the underlying associated TFs in these DARs, de novo motif analysis (Homer v4.9.1) was performed [20]. The results consistently revealed that the CTCF and CTCFL motifs were the top two significantly enriched motifs for the decreased regions but not for the increased regions (Additional file 1: Fig. S2c). Using the GREAT tool analysis, we further annotated the dynamic chromatin-accessible regions [21]. The decreased regions in HUDEP-2 were enriched in regulating the ROS biosynthesis process, cellular signaling, kinase activity, and metabolism pathways (Additional file 1: Fig. S2d; Additional file 5: Table S4). The decreased regions in HEL were also enriched in regulating the ROS biosynthesis process, cellular signaling, kinase activity, and metabolism pathways (Additional file 1: Fig. S2e; Additional file 5: Table S4). In addition, by integrating ATAC-seq and CTCF ChIP-seq datasets, the CTCF occupancy signals were more robust at the decreased DARs than those at increased DARs or control regions (Fig. 2e). Thus, these results indicate that CTCF is selectively required for maintaining the proper chromatin accessibility in the erythroid progenitor cells.

### CTCF is required for erythroid gene activation in a stage-dependent manner

**Fig. 3:**
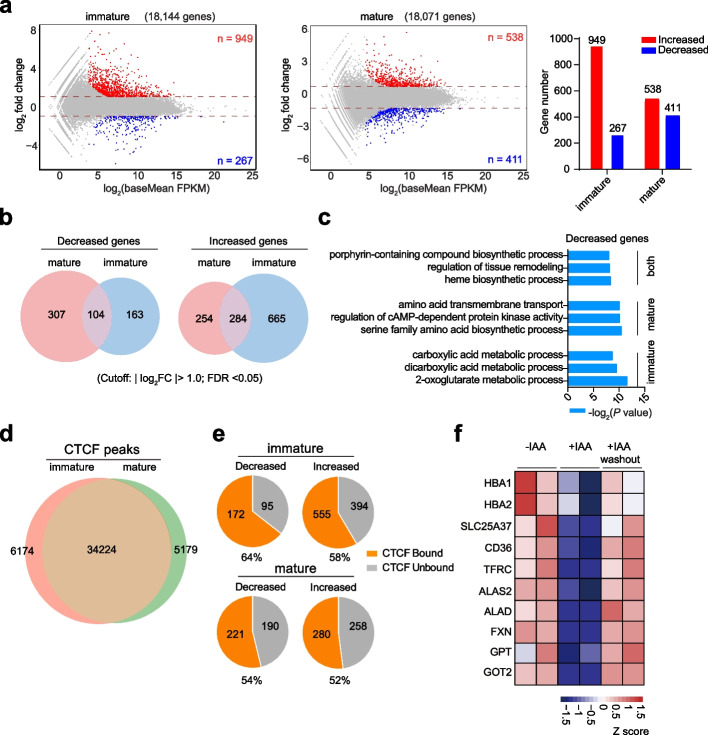
CTCF is required for target gene activation in a stage-dependent manner. **a** Volcano plot showing transcriptome changes in the CTCF-AID clones of HUEDP-2 without and with IAA treatment in an immature expansion state (left panel). Volcano plot showing transcriptome changes in two individual clones of HUEDP-2 on day 2 after induction of erythroid maturation without and with IAA treatment (medium panel). The number of differentially expressed genes in immature and mature HUDEP-2 cells before and after IAA treatment is summarized (right panel). The cutoff was based on an FDR < 0.05 and fold change > 2. **b** Overlap analysis of decreased genes between mature and immature states at the transcriptional level after CTCF depletion (left panel); and overlap analysis of increased genes between mature and immature states at the transcriptional level after CTCF depletion (right panel) in CTCF-AID HUDEP-2 cells. **c** Gene ontology analysis of the enriched biological processes associated with the genes whose expression decreased from panel **b** in the mature, immature, and both states. The relative *P* value was calculated via the EnrichR database. **d** Overlap between CTCF ChIP-seq peaks between immature and mature states. The CTCF ChIP-seq data were processed from the GSE131055 dataset [23]. **e** Percentages of CTCF occupancy in decreased and increased genes within their promoters in the immature (upper panel) and mature (lower panel) states. **f** Heatmap of erythroid marker gene and metabolism pathway gene expression in CTCF-AID HUDEP-2 cells without IAA treatment, with IAA treatment for 24 h and washout of IAA for 24 h in expansion medium

To interrogate the impact of CTCF on the gene expression network, we profiled the transcriptome using the same CTCF depletion cellular models to identify target genes in different maturation stages. We compared the whole transcriptome between the CTCF wild-type (CTCF^AID^-IAA) and CTCF-depleted (CTCF^AID^ + IAA) HUDEP-2 cells before and after induced maturation. The wild-type HUDEP-2 cells were clearly distinguished from the CTCF-depleted cells before and after maturation using the principal component analysis (PCA) (Additional file 1: Fig. S3a). Interestingly, the CTCF-depleted cells appeared close to each other before and after maturation, indicating that the differential-expressed genes after the loss of CTCF at different stages might share common targets (Additional file 1: Fig. S3a). We next performed the differential gene expression analysis with a stringent cutoff (fold change > 2 and FDR < 0.05). Before induced maturation, 267 genes decreased while 949 genes increased expression in CTCF-depleted cells compared to the control (Fig. 3a; Additional file 6: Table S5). Upon CTCF degradation after maturation, 411 and 538 genes presented a twofold decrease and increase, respectively (Fig. 3a; Additional file 6: Table S5). Importantly, the differentially expressed genes (DEGs) before and after maturation were quite different (Fig. 3b). To further explore the gene function, we performed the gene ontology (GO) analysis for the deregulated genes using the EnrichR algorithm [22]. We found that erythropoiesis-related pathways, such as the porphyrin-containing compound biosynthetic and 2-oxoglutarate metabolic processes, were enriched in the decreased genes after CTCF depletion. In contrast, the complement receptor-mediated signaling pathway and negative regulation of cytokine production were enriched in the increased genes (Fig. 3c; Additional file 1: Fig. S3b; Additional file 6: Table S5). To further confirm whether the DEGs identified after the acute CTCF degradation are direct or indirect targets, we analyzed the publicly available ChIP-seq datasets of CTCF in both immature and mature states in HUDEP-2 cells (GSE131055) [23]. About 40,371 and 39,403 reproducible peaks were called before and after maturation, respectively. Notably, over 90% of the peaks (34,224) overlapped between the two states (Fig. 3d; Additional file 7: Table S6). We then analyzed the CTCF occupancy in the deregulated genes after CTCF loss. We observed that more than half of the decreased or increased genes were directly bound by CTCF (Fig. 3e). For example, the expression of genes related to erythroid maturation (e.g., *HBA1, HBA2, TFRC, SLC25A37*, and *CD36*) and genes involved in metabolic pathways (e.g., *ALAS2, ALAD, FXN, GPT*, and *GOT2*) decreased after the induction of CTCF degradation (Fig. 3f). These results are consistent with the observation that CTCF-depleted HUDEP-2 cells underwent abnormal erythroid maturation as described previously. We also performed a transcriptome analysis of the CTCF-AID HEL cell line before and after hemin-induced maturation. We observed similar stage-dependent differences in the DEGs after CTCF depletion in the HEL model (Additional file 1: Fig. S3c and S3d; Additional file 8: Table S7). Similarly, erythroid-related targets in the sulfur compound metabolic pathway were decreased in HEL cells before and after maturation (Additional file 1: Fig. S3e; Additional file 8: Table S7). To further rule out the potential secondary effects of CTCF depletion on transcriptome changes, we performed bulk RNA-seq after CTCF degradation for shorter periods, including 6 (T6) and 12 (T12) upon CTCF depletion in the two CTCF-AID cellular systems. In total, 241 and 432 DEGs were identified for T6 and T12, respectively, in the CTCF-depleted HUDEP-2 cells (Additional file 1: Fig. S3f). Similarly, 246 and 443 differentially expressed genes were identified for T6 and T12, respectively, in the CTCF-depleted HEL cells (Additional file 1: Fig. S3f). Notably, the downregulated and upregulated genes dramatically overlapped at each time point (Additional file 1: Fig. S3g). Thus, these results suggest that CTCF is required for the expression of different subsets of genes in different maturation stages during human erythropoiesis.

### Interrogate CTCF target genes based on multi-omics integrative analysis

**Fig. 4:**
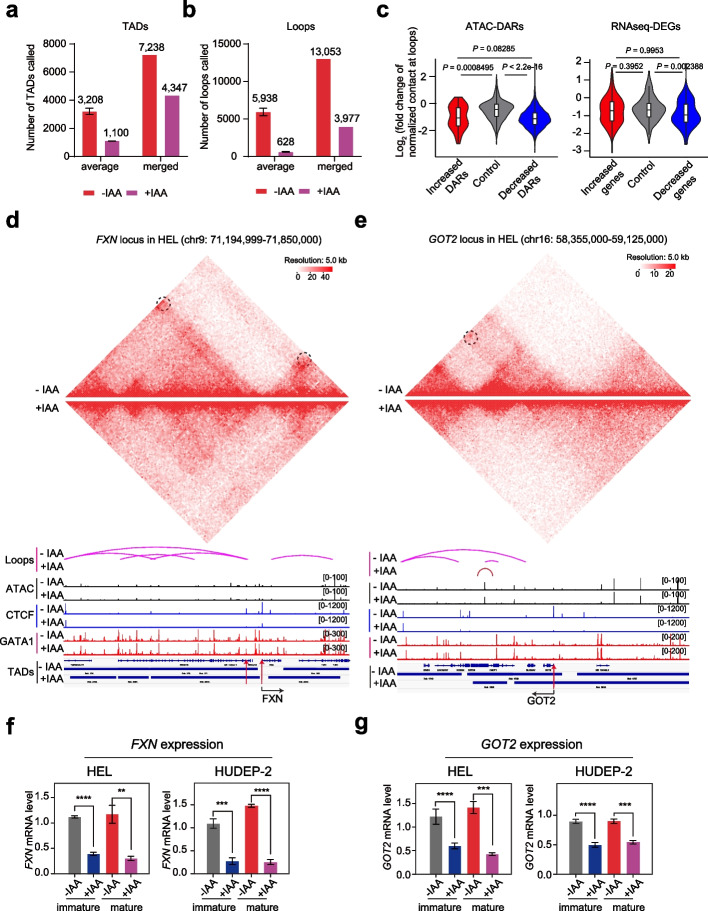
Interrogate CTCF target genes based on multi-omics integrative analysis. **a** The number of TADs called from CTCF-AID HEL cells is shown with or without 24 h of IAA treatment. The reproducibility of the Hi-C data from two independent replicates was high, allowing the integration of raw Hi-C data to recall TADs. **b** The number of chromatin loops called from CTCF-AID HEL cells is shown with or without 24 h of IAA treatment. The reproducibility of the Hi-C data from two independent replicates was high, allowing the integration of raw Hi-C data to recall loops. **c** Violin plot showing the relative fold change in normalized contact numbers from Hi-C in CTCF-AID HEL cells (+ IAA versus − IAA) at loops grouped by whether the loop anchors overlapped the DARs from ATAC-seq (left panel) or the DEGs from RNA-seq relative to their corresponding control groups. *** *P* < 0.001; **** *P* < 0.0001, according to the Wilcoxon test. **d** Screenshot of the metabolism-related gene example *FXN*, with ATAC-seq, CTCF ChIP-seq, and GATA1 ChIP-seq signals and annotated TAD domains and chromatin loops from the genome-wide Hi-C interaction map before and after IAA treatment in CTCF-AID HEL cells. The red arrow highlights the decreased ATAC-seq and CTCF ChIP-seq peaks in the enhancer and promoter regions. The black cycle indicates the position of loops identified with lost contacts after IAA treatment in CTCF-AID HEL cells. **e** Screenshot of the metabolism-related gene example *GOT2*, with ATAC-seq, CTCF ChIP-seq, and GATA1 ChIP-seq signals and annotated TAD domains and chromatin loops from the genome-wide Hi-C interaction map before and after IAA treatment in CTCF-AID HEL cells. The red arrow highlights the decreased ATAC-seq and CTCF ChIP-seq peaks in the promoter region. The black cycle indicates the loop position identified with lost contacts after IAA treatment in CTCF-AID HEL cells. **f** In both the immature and mature states, the validated mRNA level of *FXN* expression in the CTCF-AID HUDEP-2 and HEL cells was obtained without and with IAA treatment. The mRNA level was determined relative to that of β-actin from three replicates. ** *P* < 0.01, *** *P* < 0.001, **** *P* < 0.0001, unpaired Student’s *t* test. **g** In both the immature and mature states, the validated mRNA level of *GOT2* expression in the CTCF-AID HUDEP-2 cells and HEL cells without and with IAA treatment. The mRNA level was determined relative to that of β-actin from three replicates. ** *P* < 0.01; *** *P* < 0.001, **** *P* < 0.0001, unpaired Student’s *t* test

To project our observations in three-dimensional chromatin niche in human erythroid progenitor cells, we utilized in situ Hi-C to quantify the genome-wide DNA contacts via proximity ligation and next-generation sequencing in CTCF-AID HEL cells before and after 24 h of IAA treatment. A high-resolution Hi-C profiling at 5 kb showed a strong correlation between replicates (Additional file 1: Fig. S4a). The high-order chromosome folding at the resolution of A/B compartments was similar before and after the CTCF depletion in HEL cells (Additional file 1: Fig. S4b and S4c). Next, based on two replicates of our Hi-C data, we called an average of 3208 TADs and 1100 TADs before and after CTCF depletion in the HEL cells, respectively (Fig. 4a; Additional file 9: Table S8). To further explore the effect of the CTCF loss on chromatin loops, we combined the data from two replicates for further analysis. We obtained 13,053 chromatin loops within TAD boundaries before CTCF depletion, whereas 3977 chromatin loops remained after the 24 h of IAA treatment (Fig. 4b; Additional file 9: Table S8). In agreement with previous studies in other model systems [17], our Hi-C data suggests that CTCF is essential for maintaining intra-TAD DNA interactions and delineating TAD boundaries but is dispensable for higher-order compartment integrity.

To decipher the role of CTCF in gene transcriptional regulation at the 3D level, we performed multi-omics integrated analysis by utilizing ATAC-seq and RNA-seq together with the in situ Hi-C data. First, we asked whether the DARs from ATAC-seq or the DEGs from RNA-seq were directly associated with the dynamic chromatin loops. We separated the chromatin loops into three DARs groups and plotted their normalized chromatin contact numbers with different criteria (Knight-Ruiz normalization) according to previous methods [18]. Importantly, the number of lost contracts at loops overlapping the decreased DARs was significantly more than that of the loops overlapping the control regions (Wilcoxon test *P* value < 2.2 × 10^−16^) (Fig. 4c). Similarly, compared with the control genes, the decreased genes were observed with a significantly higher frequency with lost looping (Wilcoxon test *P* value = 0.002388) (Fig. 4c). These results suggest that the loss of chromatin looping after CTCF depletion was strongly correlated with the loss of chromatin accessibility and the suppression of gene transcription. Next, we explored whether these DARs or DEGs were also associated with the TAD boundaries. Accordingly, we called high-confidence TAD boundaries in the control and CTCF-depleted cells from the Hi-C dataset. Notably, the decreased or increased DARs or relative control regions were observed to have a similar distribution pattern of distance to the TAD boundaries. In contrast, only the decreased genes were more distal from the TAD boundaries than the increased or control genes were (Additional file 1: Fig. S4d). These results indicate that CTCF-mediated chromatin looping, not TAD maintenance, is correlated with chromatin accessibility and gene transcription.

The other unique application for the multi-omics data is to reveal the downstream gene regulation of CTCF. To further identify direct CTCF targets, we reasoned that direct CTCF occupancy likely affects chromatin accessibility and transcription. Therefore, we conducted an integrated data analysis using the ATAC-seq, RNA-seq, and CTCF ChIP-seq data. We selected all decreased ATAC peaks within 100 kb of the transcription start sites (TSSs) and then annotated those peaks to the associated genes. Among the deregulated genes in the immature state, 141 genes contained ATAC peaks that were decreased upon CTCF depletion. In this gene set, 46 genes were downregulated (activated by CTCF), and 95 genes were upregulated (repressed by CTCF) (Additional file 1: Fig. S4e; Additional file 10: Table S9). Moreover, over 90% of those genes were bound by CTCF in HUDEP-2 cells, identifying them as direct CTCF targets (Additional file 10: Table S9). Importantly, the GO analyses of downregulated genes with reduced ATAC peaks upon CTCF depletion revealed an enrichment of genes associated with the amino acid metabolic, sulfur compound metabolic, and glycoprotein biosynthetic processes (Additional file 1: Fig. S4f; Additional file 11: Table S10). For example, CTCF exhibited strong occupancy at the loci of three direct target genes that are essential for mitochondrial heme and iron-sulfur cluster metabolism (e.g., *FXN* and *GOT2*) and novel RNA-binding protein (*RBM45*), of which the mRNA levels and the chromatin accessibility were significantly decreased, accompanied by loss of the chromatin looping after the induction of CTCF degradation (Fig. 4d and 4e; Additional file 1: Fig. S5a-d). All three gene expression patterns were confirmed and validated by Q-PCR after the CTCF depletion in the HEL and HUDEP-2 cells (Fig. 4f, g; Additional file 1: Fig. S5e). Among them, *RBM45* is one of the most sensitive downstream targets of CTCF, and the CTCF protein binds directly to the promoter region of *RBM45*. The CTCF binding site in the *RBM45* promoter is functional for gene regulation, supported by the fact that CRISPR/Cas9-mediated disruption of this site significantly reduced its mRNA level (Additional file 1: Fig. S5f). Thus, these results suggest that CTCF can directly bind and activate its target genes through chromatin accessibility and chromatin looping in erythroid lineage.

### CTCF is also required for a subset of GATA1-mediated gene repression

**Fig. 5:**
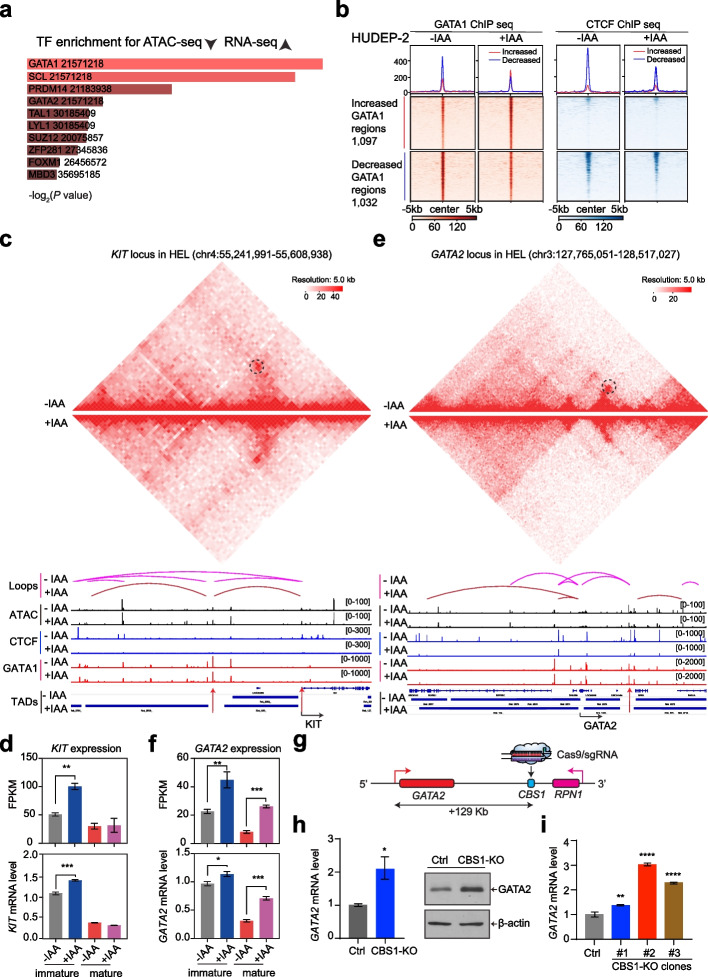
CTCF is also required for a subset of GATA1-mediated gene repression. **a** The top ten enriched TFs associated with the genes with decreased ATAC peaks and upregulated expression in IAA-treated cells were identified via the EnrichR database. The relative *P* value was calculated from the database. **b** Heatmaps showing the dynamic GATA1 binding signal centered at the GATA1 peak summit for decreased and increased GATA1 regions in the HUDEP-2 cell line without and with IAA treatment for 24 h. The GATA1 ChIP-seq results were merged with analyses from two independent replicates. **c** Screenshot of *KIT*, with ATAC-seq, CTCF ChIP-seq, and GATA1 ChIP-seq signals and annotated TAD domains and chromatin loops from the genome-wide Hi-C interaction map before and after IAA treatment in CTCF-AID HEL cells. The red arrow highlights the decreased CTCF ChIP-seq peaks in the enhancer and promoter regions. The black cycle indicates the loop position identified with no change before and after IAA treatment in CTCF-AID HEL cells. **d** Plot of fragments per kilobase of peaks per million reads mapped (FPKM) values and validated mRNA levels of *KIT* expression in CTCF-AID HUDEP-2 cells without and with IAA treatment in both the immature and mature states. The mRNA level was determined relative to that of β-actin from three replicates. ** *P* < 0.01, *** *P* < 0.001, unpaired Student’s *t* test. **e** Screenshot of *GATA2*, with ATAC-seq, CTCF ChIP-seq, and GATA1 ChIP-seq signals and annotated TAD domains and chromatin loops from the genome-wide Hi-C interaction map before and after IAA treatment in CTCF-AID HEL cells. The red arrow highlights the decreased ATAC-seq and CTCF ChIP-seq in the distal region. The black cycle indicates the loop position identified in CTCF-AID HEL cells. **f** Plot of fragments per kilobase of peaks per million reads mapped (FPKM) values and validated mRNA levels of *GATA2* expression in CTCF-AID HEL cells without and with IAA treatment in both the immature and mature states. The mRNA level was determined relative to that of β-actin from three replicates. * *P* < 0.05, ** *P* < 0.01, *** *P* < 0.001, unpaired Student’s *t* test. **g** The cartoon illustrating the location of CBS1 (CTCF binding site 1) between *GATA2* and its downstream neighbor gene *RPN1*, which is about 129 kb to the first exon of the GATA2 gene. **h** Measurement of *GATA2* mRNA by quantitative real-time PCR (left panel) and GATA2 protein by Western blot (right panel) in the GATA2-CBS1-sgRNA targeted bulk population relative to non-targeting sgRNA control in HUDEP-2 cells. The mRNA expression levels were normalized to those of β-actin mRNA. The graph shows the results as the mean values ± SEMs from three replicates. *P < 0.05, unpaired Student’s *t* test. The β-actin was used as a loading control for immunoblots. **i** Measurement of *GATA2* mRNA by quantitative real-time PCR in three individual GATA2-CBS1 knockout HUDEP-2 cell clones generated with Cas9 + CBS1-sgRNA. The expression levels were normalized to those of β-actin mRNA. The graph shows the results as the mean values ± SEMs from three replicates. **P < 0.01, **** *P* < 0.0001, unpaired Student’s *t* test

Importantly, we observed that a proportion of the subset of upregulated genes was associated with the ATAC peaks that were reduced upon CTCF depletion (Additional file 1: Fig. S4e). The GO analysis of those upregulated genes revealed an enrichment of genes associated with positive regulation of cytokine production, muscle system processes, negative regulation of transport, epithelial cell proliferation, and ameboidal-cell type migration (Additional file 1: Fig. S4f; Additional file 11: Table S10). These genes were most likely repressed by CTCF, suggesting the role of CTCF in suppressing alternative lineage genes. More importantly, by analyzing the potential transcription factor occupancy upstream of those genes, hematopoietic transcription factors, including GATA1, were identified as the most significant TFs (Fig. 5a; Additional file 11: Table S10). These results suggest that CTCF may coordinate with GATA1 to suppress a subset of genes.

The well-known erythroid “master” transcription factor GATA1 controls the activation of the erythroid-specific gene signatures and the suppression of other lineage genes. Although CTCF colocalizes with GATA1 during human erythropoiesis, how CTCF affects the occupancy of genome-wide GATA1 remains unknown. We hypothesized that the acute CTCF depletion may disrupt a specific subset of GATA1 targets. To test this hypothesis, we further profiled the genome-wide occupancy of GATA1 via ChIP-seq before and after the CTCF depletion. First, we called 49,114 and 57,071 reproducible GATA1 peaks in the CTCF-AID HUDEP-2 and HEL cells before the IAA treatment, and the GATA motifs were significantly enriched in the reproducible peaks (Additional file 12: Table S11). We then compared the dynamic GATA1 binding signals before and after the CTCF depletion. Notably, 1032 decreased and 1097 increased GATA1 regions were identified in the HUDEP-2 cells, while 1902 decreased and 954 increased GATA1 regions were identified in the HEL cells (Fig. 5b; Additional file 1: Fig. S6a). Both CTCF and GATA1 binding signals were significantly reduced after the CTCF depletion for the decreased GATA1 regions, and those two TFs were identified among the top significantly enriched TFs by Homer analysis (Additional file 1: Fig. S6b), suggesting that CTCF is required for the proper occupancy of GATA1 in a subset of regions. Moreover, the genomic distribution of the decreased GATA1 was located mainly in the distal regulatory regions (Additional file 1: Fig. S6c). We further explored whether the DEGs were correlated with reduced GATA1 occupancy at the nearby locus. A more significant correlation between increased genes and decreased GATA1 occupancy was identified in HUDEP-2 cells (Additional file 1: Fig. S6d). Taken together, these results suggest that a proportion of the reduced GATA1 occupancy might correlate with partial gene upregulation after CTCF depletion.

Furthermore, we validated some of the GATA1-suppressed targets. For example, CTCF exhibited strong occupancy at the *KIT* locus, in which the mRNA expression was significantly increased while the chromatin looping remained unchanged in the immature stage after the induction of CTCF degradation (Fig. 5c, d; Additional file 1: Fig. S6e). KIT is critical for the cell proliferation of both HSCs and erythroid progenitors and is also a well-known direct target gene of both GATA1 and GATA2 that needs to be repressed after erythroid maturation [24]. Next, we determined how CTCF changed the expression level and chromatin accessibility of both GATA1 and GATA2. Strikingly, we found that *GATA2* mRNA significantly increased after depletion of CTCF in the HUDEP-2 line before and after erythroid maturation (Fig. 5e, f).

To decipher the CTCF functional occupancy site, we analyzed it more deeply by combining all the chromatin accessibility, histone modification, CTCF, and GATA1 occupancy datasets across the annotated TAD region in different cellular contexts. Notably, the *GATA2* locus was directly bound by CTCF at multiple sites, mainly located in distal regions. In particular, two CTCF binding sites in the *GATA2* distal enhancer region exhibited significantly decreased chromatin accessibility after the CTCF depletion in the HEL and HUDEP-2 lines with opposite orientations in the nearest downstream region of *RPN1* (Fig. 5e; Additional file 1: Fig. S6f). Of note, the Hi-C data collected from both HUDEP-2 and HEL cells confirmed the interaction between the *GATA2* promoter region and the left-orientated CTCF binding site 1 (CBS1) (Fig. 5e; Additional file 1: Fig. S6f). Although CTCF binds directly to the *GATA2* locus in multiple human cell contexts (Additional file 1: Fig. S7a), its suppressive role is not conserved. We re-analyzed the regulation using SEM, representing the human B-ALL cell context (Additional file 1: Fig. S7b). Upon CTCF degradation, the ATAC-seq and CTCF binding were lost at the *GATA2* locus (Additional file 1: Fig. S7b). However, the genes in the *GATA2* locus exhibited no decrease or a mild decrease in expression after the CTCF loss (Additional file 1: Fig. S7c). To determine the role of the CTCF binding site (CBS) in the *GATA2* locus, we performed the following experiments to explore its regulatory role. To further validate the function of the 3’ distal element, we used the CRISPR/Cas9 system to delete the CTCF binding motif. A sgRNA targeting the CBS1 in the distal chromatin accessibility region achieved an overall indel frequency of 70% in the targeted pool population (Fig. 5g; Additional file 1: Fig. S7d). Importantly, disruption of the CBS1 in the distal region of GATA2 led to a significant increase in *GATA2* expression but a decrease for the neighbor gene *RPN1* in the bulk population and generated single knockout clones compared with the non-targeting guide RNA control (Fig. 5h, i; Additional file 1: Fig. S7e and S7f). Thus, the CBS1 in the distal region acts as a transcription repressive element for GATA2 expression in erythroid progenitor cells.

## Discussion

In this study, we systematically characterized the CTCF-controlled human erythroid transcriptome network under a three-dimensional chromatin niche using the novel AID system. Although CTCF is a well-known master regulator of multiple types of cell differentiation [25], deciphering its precise role is challenging, as the loss of its function often causes severe cell growth defects. The AID system demonstrates its unique advantage in studying the immediate role of CTCF in genome organization and transcriptional regulation. Specifically, we revealed the role of CTCF in erythroid maturation after the induction of CTCF degradation. We observed an erythroid maturation defect after differentiation, as indicated by a higher level of the later maturation cell surface marker BAND3, slower proliferation, and less hemoglobinization (Fig. 1; Additional file 1: Fig. S1). This abnormal phenotype in the HUDEP-2 line, with a higher percentage of BAND3^+^ cells and less hemoglobinization, is consistent with previous observations of the disruption of one CTCF binding site around its downstream target gene, *RBM38* [23]. However, the erythroid progenitor cell line HUDEP-2 maintained quite a normal expansion state after the induction of CTCF depletion, suggesting a stage dependency of CTCF during erythroid maturation. Similarly, ectopic expression of CTCF in the K562 cell line accelerated erythroid differentiation by sacrificing cell growth. In contrast, the knockdown of CTCF by RNAi inhibited erythroid differentiation but increased cell proliferation [13], suggesting an antagonistic role of CTCF between cell proliferation and differentiation. With the availability of a new version of the AID system in a mouse model [26], it would be ideal for deciphering the role of CTCF in erythropoiesis and hematopoiesis in vivo in the future.

Several previous studies demonstrated the function of CTCF upon auxin-mediated degradation in mouse cell models, providing evidence that the maintenance of topological boundaries is critical for appropriate gene expression [27–29]. However, much less is known about gene regulation during the induction of CTCF degradation in the human cellular context. Our integrative multi-omics analysis also confirmed that the decreased DARs and genes were significantly associated with the loss of chromatin loops. Our work identified hundreds of deregulated genes, of which over half were directly bound by nearby CTCF (Fig. 3). Our functional study demonstrated that acute degradation of CTCF affected the gene signature to a greater extent in a human model than in the mouse erythroblast cell line G1E-ER4, which exhibited a mild effect on gene expression after the short-term scale CTCF depletion [28]. Compared with those in the mouse context, gene proportions may be due to differences in the auxin exposure timescale, technology resolution, and species dependency. Our observation that CTCF controls different erythroid gene subsets before and after maturation suggests that CTCF plays a dynamic role in lineage differentiation. Thus, it might be essential to consider context specificity and the exact differentiation status when CTCF regulates gene expression to control cell fate.

**Fig. 6:**
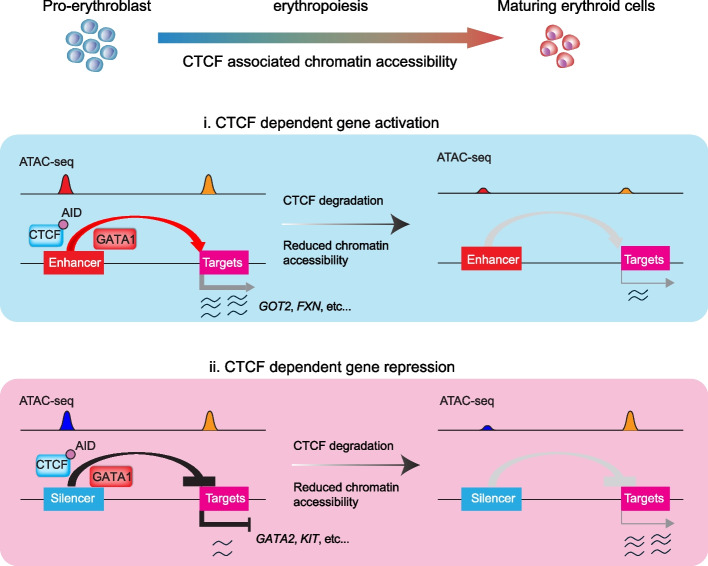
Proposed model for CTCF-dependent gene regulation in erythropoiesis. Our study identified two subsets of genes with reduced chromatin accessibility after CTCF depletion, suggesting a simplified model with two different patterns of CTCF-dependent gene regulation in the context of erythroid cells. (*i*) CTCF-dependent gene activation is associated with decreased chromatin accessibility and chromatin looping. Upon erythroid maturation, CTCF is required to activate the expression of a subset of erythroid lineage genes, such as *FXN* and *GOT2*, which are involved in cellular amino acid and heme metabolism. (*ii*) CTCF-dependent gene repression with dynamic GATA1 occupancy. Upon erythroid maturation, CTCF is required to repress the expression of a subset of nonerythroid lineage genes, such as *GATA2* and *KIT.* These genes are also enriched with a reduction in GATA1 occupancy, suggesting that CTCF is required for a subset of GATA1-mediated gene repression

Our study also identified two subsets of genes with reduced chromatin accessibility after the CTCF depletion, suggesting that at least two different models of the CTCF-dependent gene regulation are involved in erythroid lineage specification (the proposed model in Fig. 6). Chromatin topologically associating domains are crucial in precisely controlling developmental regulators, including the recently identified factor RUNX1 in hematopoiesis [14, 30–32]. Interestingly, GATA1 also harbors an insulator site occupied by CTCF [32]. Our study here sheds new light on the role of CTCF in suppressing a subset of target genes that coordinate with the master erythroid transcription factor GATA1. To further support this observation, significantly decreased GATA1 binding regions were identified, and motif enrichment analysis revealed that both the CTCF and GATA1 motifs were significantly enriched (Fig. 5). This mechanism was further illustrated by two well-known GATA1-repressed genes, *KIT* and *GATA2*, involved in erythropoiesis [33, 34]. This model is simplified as numerous increased genes were observed without gain of GATA1 reduction or with loss of GATA1, requiring the full consideration of other TFs or corepressors [35]. Although many studies have aimed to identify the cis-regulatory element in the *GATA2* locus [36], there is limited knowledge on the suppression of GATA2 during erythropoiesis. Our work illustrates the suppressive role of CTCF in GATA2 expression via a distal chromatin-accessible CTCF binding site. Moreover, our study revealed a subset of target genes directly repressed by CTCF in erythroid cells, including *KIT* and other genes involved in cytokine production. Notably, numerous CTCF-suppressed genes were enriched with GATA1 occupancy, suggesting the coordination of CTCF with GATA1. Especially in erythropoiesis, GATA1 mediates the displacement of GATA2 from chromatin at the distal region and represses GATA2 and KIT expression; this paradigm is called the GATA switch [33, 37]. The GATA2 expression level is positively regulated by various cis-regulatory elements, including distal enhancers [36]. Nevertheless, it remains unknown whether sequences extending beyond the core enhancer contribute to GATA2 expression, especially insulators or silencers that mediate GATA2 suppression during erythroid differentiation. This study, together with our functional validation, suggested the inhibitory role of the suppressive element in GATA2. However, to fully understand the regulatory role of the TAD boundary, additional studies are necessary to disrupt the multiple CTCF sites across the TAD and validate their effects on the expression of GATA2 and other nearby genes and the chromatin accessibility and architecture confirmation consequences. The newly identified repressive role of CTCF might have a significant function in blood development and related blood disorders, which needs further characterization.

## Conclusions

In summary, we have established novel CTCF-AID cellular tools in human erythroid lineage and characterized the immediate transcriptional network with inducible degradation of endogenous CTCF in human erythropoiesis. We observed a unique feature of CTCF in selectively maintaining the proper chromatin openness in the erythroid lineage, with thousands of chromatin accessibility regions reduced after the depletion of CTCF. Moreover, we identified hundreds of erythroid genes controlled by CTCF, including activated and repressed targets with reduced chromatin accessibility. These results in the cell model from this study will potentially impact the transcriptional regulation of CTCF in both development and blood cancers. More importantly, we are confident that the high-quality multi-omics data collected from CTCF-AID erythroid cell models will serve as valuable resources for the research community.

## Methods

### Culture and induced maturation of HUDEP-2 and HEL cells

Mycoplasma-free HUDEP-2 cells were cultured as described previously [15]. The immature cells were expanded in StemSpan serum-free medium (SFEM; STEMCELL Technologies) supplemented with 1 µM dexamethasone, 1 µg/mL doxycycline, 50 ng/mL human stem cell factor (SCF), 3 units/mL erythropoietin (EPO), and 1% penicillin–streptomycin. To induce erythroid maturation, HUDEP-2 cells were cultured in a differentiation medium composed of IMDM base medium (Invitrogen) supplemented with 2% FBS, 3% human serum albumin, 3 units/mL EPO, 10 µg/mL insulin, 1000 µg/mL holo-transferrin, and 3 units/mL heparin. To achieve auxin-induced degradation, two single-cell derived clones were treated using an expansion medium or differentiation medium supplemented with 500 µM IAA (natural auxin) (Sigma) for 24 h to induce the degradation of CTCF. The “wash out” cells were prepared from treated cells centrifuged and resuspended in PBS three times, followed by an additional culture for 24 h in a regular medium. HEL cells were maintained in RPMI-1640 medium (Corning, 10–040–CV) supplemented with 10% fetal bovine serum (FBS) (HyClone), and erythroid maturation was induced by the addition of 30 µM hemin (MCE, HY-19424) for 48 h. HUDEP-2 cells were provided by Ryo Kurita and Yukio Nakamura (Cell Engineering Division, RIKEN BioResource Center, Tsukuba, Japan). The HEL cell line was obtained from the Cell Resource Center, Peking Union Medical College (PCRC). Before the experiments were performed, the identity of cell lines was verified via short tandem repeat (STR) analysis, and the cells were confirmed to be mycoplasma-negative.

### CRISPR/Cas9-mediated genome editing in HUDEP-2 and HEL cells

The sgRNA sequences targeting CTCF binding sites (CBSs) were selected and designed based on the CTCF motif annotation from CTCF ChIP-seq peaks and generated as oligonucleotides. After annealing, the constructs were cloned and inserted into the *Bsm*BI site of the pXPR_003 vector (Plasmid #52963, Addgene). For cell pool genome editing, HUDEP-2 cells stably expressing Cas9 were transduced with a lentiviral vector (pXPR_003) encoding individual single guide RNAs (sgRNAs). The cells were incubated for 7–10 days with 10 µg/mL blasticidin and 1 µg/mL puromycin to select for transduction with the sgRNA and Cas9 vectors, respectively. On-target insertion/deletion mutations were characterized by PCR amplification, followed by Sanger sequencing, and then analyzed via TIDE [38] or ICE. For miniAID-mClover3 knock-in delivery, 2.5 μg of the donor plasmid and 2.5 μg of the all-in-one Cas9-gRNA plasmid were used for 5 million HEL cells. The HEL cells were electroporated using the Neon™ NxT Electroporation (Neon) with the Neon™ Transfection Kit (Invitrogen by Thermo Fisher Scientific, MPK1096B). Twenty-four hours after electroporation, the cells were sorted for the expression of the GFP fluorescent marker to enrich the knock-in cell population. After the sorted cells recovered in culture for up to 3 weeks, a second sort was performed to select cells for successful knock-in by sorting for cells expressing the knock-in GFP fluorescent marker. Two weeks later, a third sort was repeated to identify single-cell-derived clones based on the high GFP expression. The primers and sequences used for PCR and the sgRNAs used are listed in Additional file 13: Table S12.

### Flow cytometry

Erythroid differentiation and maturation were monitored via flow cytometry using fluorescein isothiocyanate (FITC)-conjugated anti-CD235 (BD Biosciences, clone GA-R2, catalog #561017), allophycocyanin (APC)-conjugated anti-BAND3 (homemade), and violet blue-conjugated anti-CD49d (Miltenyi Biotec, clone MZ18-24A9, catalog #130–099–680) antibodies.

### Quantitative real-time PCR

RNA from approximately 0.5–1 × 10^6^ cells was extracted with an RNeasy Kit (Invitrogen). Reverse-transcription reactions were performed with random hexamers by using iScript (Bio-Rad). The mRNA expression levels of all the genes were quantified using SYBR Green real-time PCR. The primers were obtained from IDT and are listed in Additional file 13: Table S12.

### Western blot analysis

The cells were suspended in Pierce IP Lysis Buffer (Thermo Fisher Scientific, catalog #87787) supplemented with 1 mM phenylmethylsulfonyl fluoride and a 1:500 protease inhibitor cocktail (Sigma-Aldrich). Proteins were resolved on polyacrylamide gels (Bio-Rad), transferred to a PVDF membrane, and incubated in a blocking buffer (5% nonfat milk in TBST). Antibody staining was visualized via an Odyssey CLx Imaging System. The secondary antibody (goat anti-rabbit IgG [H + L] with HRP; Invitrogen, catalog #31460) was diluted 1:2000 in blocking buffer (to a final concentration of 0.4 µg/mL). All the primary antibody information is listed in Additional file 13: Table S12.

### RNA-seq and data analysis

For RNA-seq, RNA was extracted from 5 × 10^6^ HUDEP-2 and HEL cells (with at least three biological replicates in each case) by using an RNeasy Mini Kit (Qiagen) according to the previous protocol [1]. Briefly, a TruSeq Stranded mRNA Library Prep Kit (Illumina) was used to enrich polyA + RNA and create libraries for sequencing using a HiSeq2000 System (Illumina). The RNA-seq reads were aligned to the hg19 human genome using the StrongARM pipeline [39]. The HTSeq-count script (HTSeq framework, version 0.6.1p1) was used to obtain per-gene counts based on GENCODE annotation [40]. Hemoglobin and small nucleolar RNAs were excluded from the analysis. All differentially expressed transcripts were selected using a cutoff of *P* < 0.05.

### ATAC-seq and data analysis

The ATAC-seq library was prepared according to the published omni-ATAC protocol [41]. For the HUDEP-2 cells, 50,000 live cells were used per sample [1]. After centrifugation at 500 rpm for 5 min at 4 °C (Eppendorf 5417R refrigerated centrifuge), the cell pellets were resuspended in cold lysis buffer supplemented with protease inhibitors (10 mM Tris with a pH 7.4, 10 mM NaCl, 3 mM MgCl_2_, and 0.1% IGEPAL), followed by centrifugation. The pellets were resuspended in 25 μL of tagment DNA buffer (Nextera, FC-121–1030) and used directly in the transposition reaction. Nextera Tn5 (Nextera, FC-121–1030) was added to the resuspended nuclei, and the transposition reaction mixture was incubated at 37 °C for 30 min. After transposition, the DNA was purified using a Qiagen MinElute PCR purification kit (Qiagen, 28,004). Indexing PCR was conducted for 12 cycles using the NEBNext HiFi 2X PCR Master Mix (NEB, M0541S) and indexing primers. The PCR products were purified at a 1:3 ratio of Agencourt AMPure XP beads (Beckman Coulter, A63881). Libraries were paired-end 100-bp sequenced using an Illumina HiSeq 4000 system. The ATAC-seq datasets were analyzed via the previous methods [18].

### ChIP-seq data analysis

The ChIP experiments were performed as previously described [42], with at least two biological replicates for each study. First, 2 × 10^7^ HUDEP-2 and HEL cells were suspended in 50 mL of PBS and processed according to a previously detailed method [1]. The quality of the ChIP-seq library was determined with an Agilent 2100 Bioanalyzer using a high-sensitivity chip (Agilent). The average sizes of the ChIP-seq libraries ranged from 350 to 600 bp. For multiplexing, equal molar quantities of libraries were combined by considering the sequencing depth per sample (40 million reads per library). The ChIP-seq libraries were sequenced via an Illumina HiSeq2500 system or a NextSeq platform with single-end reads of 50 bases. Peaks were called twice for each sample with FDR-corrected *P*-value cutoffs of 0.05 (the high confidence peak) and 0.5 (the low confidence peak), and reproducible peaks were finalized by requiring a peak called as high confidence in one sample and low confidence in any other replicate. For visualization, reads were extended to the fragment size estimated by the SPP and normalized to 15 M nonduplicated reads to generate bigwig files. Deeptools (v2.5.7) was used to plot the average heatmap in 10-bp bins.

### Hi-C and integrative data analysis

In situ Hi-C experiments were conducted as previously described [43]. Briefly, five million CTCF-AID knock-in HEL cells without or with IAA treatment for 24 h were crosslinked with 1% formaldehyde for 10 min at room temperature, digested with 125 units of MboI, labeled with biotinylated nucleotides and ligated for proximity ligation. After decrosslinking, the ligated DNA was purified and sheared to 300–500 bps. Ligation junctions were pulled down with streptavidin beads and prepared as a standard Illumina library. Each library underwent 75 cycles of paired-end sequencing on the Illumina HiSeq 4000 system. The Hi-C dataset and the integrative multi-omics analysis were performed using previous methods [17, 18]. Each sample had two replicates, and we merged the replicates after confirming reproducibility. Each sample contained > 250 million paired-end fragments, > 60% ligation motif present, and > 200 million contacts.

### Software and statistical analysis

Prism software and R were used for data processing, statistical analysis, and result visualization (http://www.graphpad.com). The R language and environment for graphics (https://www.r-project.org) were used for the bioinformatics analysis of the ATAC-seq and RNA-seq data. The package Limma-voom was used for differential gene expression analysis from RNA-seq and DEseq2 for the differential chromatin accessibility analysis from ATAC-seq datasets. The R packages used for all analyses described in this manuscript were from Bioconductor and CRAN. A two-tailed, unpaired Student’s *t* test was used for comparisons between the two groups. The graphs’ bars represent the standard error of the mean (SEM), as indicated in the figure legends. *P* < 0.05 was considered statistically significant for all figures; * indicates *P* < 0.05, ** indicates* P* < 0.01, *** indicates *P* < 0.001, and **** indicates *P* < 0.0001. Gene Ontology and TF enrichment analyses were conducted according to the default parameters in their native implementations using EnrichR [22]. Fisher’s exact test was used for the statistical enrichment of gene lists. No statistical methods were used to predetermine the sample size.

## Supplementary Information

Additional file 1: Supplementary Figures S1 – S7 and figure legend.

Additional file 2: Table S1. ATAC-seq peak data summary information from the analysis in CTCF-AID HUDEP-2 cells with IAA treatment vs. without IAA treatment grown in culture under immature conditions for 24 hours.

Additional file 3: Table S2. ATAC-seq peak data summary information from the analysis in CTCF-AID HEL cells with IAA treatment vs. without IAA treatment grown in culture under immature conditions for 24 hours.

Additional file 4: Table S3. Enrichment analysis for transcription factors in increased and decreased ATAC-seq peaks, as well as unchanged peaks in CTCF-AID HUDEP-2 cells with IAA treatment vs. without IAA treatment grown in culture under immature conditions for 24 hours. Three separate sheets include decreased vs. unchanged, increased vs. unchanged, and increased vs. decreased, respectively.

Additional file 5: Table S4. GREAT analysis for differential accessibility regionsin CTCF -AID HUDEP-2 and HEL cells with IAA treatment vs. without IAA treatment grown in culture under immature conditions for 24 hours.

Additional file 6: Table S5. RNA transcriptome analysis of CTCF-AID HUDEP-2 cells with IAA treatment vs. without IAA treatment grown in culture under immature and mature conditions for 24 hours.

Additional file 7: Table S6. CTCF ChIP-seq peaks before and after HUDEP-2 erythroid maturation from the public dataset and CTCF ChIP-seq peaks before and after IAA treatment before and after IAA treatment in both HUDEP-2 and HEL cells in the immature state.

Additional file 8: Table S7. RNA transcriptome analysis of CTCF-AID HEL cells with IAA treatment vs. without IAA treatment grown in culture under no hemin and hemin treated conditions for 24 hours.

Additional file 9: Table S8. Hi-C datasets, and annotated TADs and chromatin loops information in CTCF-AID HEL cells with IAA treatment vs. without IAA treatment grown in culture under no hemin treated conditions for 24 hours.

Additional file 10: Table S9. CTCF direct target genes from ATAC-seq and RNA-seq integrative analysis.

Additional file 11: Table S10. Gene Ontology analysis, and Transcription factors occupancy analysis for the CTCF direct target genes from ATAC-seq and RNA-seq integrative analysis.

Additional file 12: Table S11. GATA1 ChIP-seq peaks before and after IAA treatment before and after IAA treatment in both HUDEP-2 and HEL cells in the immature state.

Additional file 13: Table S12. Oligonucleotides, DNA primers, and antibodies used in this study. Oligonucleotides were used for sgRNA vector construction; DNA primers were used to analyze Cas9-mediated indels by Sanger sequencing; antibodies were used for Western blotting, immunoprecipitation, or ChIP-seq analysis.

Additional file 14. Uncropped images for the blots in figure 1b, figure 5h and supplementary figure S1a.

## Data Availability

The RNA-seq, ATAC-seq, ChIP-seq, and Hi-C raw data generated from this study were deposited into GEO under the accession number: Super-series GSE201822 [44]. The RNA-seq data from HUDEP-2 cells and CTCF Cut&Run data from SEM cells were originally deposited into GEO under the accession number: Super-series GSE174528 [17, 45]. The ChIP-seq data of normal human cell lines and cancer cell lines were obtained from ENCODE. The ATAC-seq data from the SEM line were downloaded from the GSE153237 [18, 46]. The CTCF ChIP-seq datasets in HUDEP-2 cells were obtained from the GSE131055 [23, 47]. And H3K4me3, H3K27ace and H3K4me1 ChIP-seq datasets in HUDEP-2 cells were obtained from the GSE115357 [1, 48]. The Hi-C datasets in HUDEP-2 cells were obtained from GSM4873113 [49, 50]. Code repositories collected at https://doi.org/10.6084/m9.figshare.c.6186670 included the RNA-seq analysis (https://doi.org/10.6084/m9.figshare.24803238.v1), ATAC-seq QC and peaking calling (https://doi.org/10.6084/m9.figshare.13046432.v1), Integrative analysis ChIP-seq and RNA-seq (https://doi.org/10.6084/m9.figshare.21045889.v1), Hi-C and HiChIP analysis (https://doi.org/10.6084/m9.figshare.21002533.v1) [51, 52, 56,53,54,55].

## References

[1] Xu P, Scott DC, Xu B, Yao Y, Feng R, Cheng L, et al. FBXO11-mediated proteolysis of BAHD1 relieves PRC2-dependent transcriptional repression in erythropoiesis. Blood. 2021;137:155–67. 33156908 10.1182/blood.2020007809 PMC7820877

[2] Karayel Ö, Xu P, Bludau I, Velan Bhoopalan S, Yao Y, Ana Rita FC, et al. Integrative proteomics reveals principles of dynamic phosphosignaling networks in human erythropoiesis. Mol Syst Biol. 2020;16:e9813. 33259127 10.15252/msb.20209813 PMC7706838

[3] An X, Schulz VP, Mohandas N, Gallagher PG. Human and murine erythropoiesis. Curr Opin Hematol. 2015;22:206–11. 25719574 10.1097/MOH.0000000000000134 PMC4401149

[4] Nandakumar SK, Ulirsch JC, Sankaran VG. Advances in understanding erythropoiesis: evolving perspectives. Br J Haematol. 2016;173:206–18. 26846448 10.1111/bjh.13938 PMC4833665

[5] Sankaran VG, Weiss MJ. Anemia: progress in molecular mechanisms and therapies. Nat Med. 2015;21:221–30. 25742458 10.1038/nm.3814 PMC4452951

[6] Li D, Wu F, Zhou S, Huang XJ, Lee HY. Heterochromatin rewiring and domain disruption-mediated chromatin compaction during erythropoiesis. Nat Struct Mol Biol. 2023;30:463–74. 36914797 10.1038/s41594-023-00939-3

[7] Li D, Zhao XY, Zhou S, Hu Q, Wu F, Lee HY. Multidimensional profiling reveals GATA1-modulated stage-specific chromatin states and functional associations during human erythropoiesis. Nucleic Acids Res. 2023;51:6634–53. 37254808 10.1093/nar/gkad468 PMC10359633

[8] Bi H, Hou Y, Wang J, Xia Z, Wang D, Liu Y, et al. Chromatin reconstruction during mouse terminal erythropoiesis. iScience. 2022;25:105554. 36465116 10.1016/j.isci.2022.105554 PMC9709226

[9] Oudelaar AM, Higgs DR. The relationship between genome structure and function. Nat Rev Genet. 2021;22:154–68. 33235358 10.1038/s41576-020-00303-x

[10] Takayama N, Murison A, Takayanagi S, Arlidge C, Zhou S, Garcia-Prat L, et al. The transition from quiescent to activated states in human hematopoietic stem cells is governed by dynamic 3D genome reorganization. Cell Stem Cell. 2021;28:488-501.e10. 33242413 10.1016/j.stem.2020.11.001

[11] Qi CF, Martensson A, Mattioli M, Dalla-Favera R, Lobanenkov VV, Morse HC. CTCF functions as a critical regulator of cell-cycle arrest and death after ligation of the B cell receptor on immature B cells. Proc Natl Acad Sci U S A. 2003;100:633–8. 12524457 10.1073/pnas.0237127100 PMC141048

[12] Heath H, De Almeida CR, Sleutels F, Dingjan G, Van De Nobelen S, Jonkers I, et al. CTCF regulates cell cycle progression of αβ T cells in the thymus. EMBO J. 2008;27:2839–50. 18923423 10.1038/emboj.2008.214 PMC2580790

[13] Torrano V, Chernukhin I, Docquier F, D’Arcy V, León J, Klenova E, et al. CTCF regulates growth and erythroid differentiation of human myeloid leukemia cells. J Biol Chem. 2005;280:28152–61. 15941718 10.1074/jbc.M501481200

[14] Owens DDG, Anselmi G, Oudelaar AM, Downes DJ, Cavallo A, Harman JR, et al. Dynamic Runx1 chromatin boundaries affect gene expression in hematopoietic development. Nat Commun. 2022;13:773. 35140205 10.1038/s41467-022-28376-8 PMC8828719

[15] Kurita R, Suda N, Sudo K, Miharada K, Hiroyama T, Miyoshi H, et al. Establishment of immortalized human erythroid progenitor cell lines able to produce enucleated red blood cells. PLoS One. 2013;8:e59890. 23533656 10.1371/journal.pone.0059890 PMC3606290

[16] Kuppers DA, Arora S, Lim Y, Lim AR, Carter LM, Corrin PD, et al. N6-methyladenosine mRNA marking promotes selective translation of regulons required for human erythropoiesis. Nat Commun. 2019;10:4596. 31601799 10.1038/s41467-019-12518-6 PMC6787028

[17] Hyle J, Zhang Y, Wright S, Xu B, Shao Y, Easton J, et al. Acute depletion of CTCF directly affects MYC regulation through loss of enhancer-promoter looping. Nucleic Acids Res. 2019;47:6699–713. 31127282 10.1093/nar/gkz462 PMC6648894

[18] Xu B, Wang H, Wright S, Hyle J, Zhang Y, Shao Y, et al. Acute depletion of CTCF rewires genome-wide chromatin accessibility. Genome Biol. 2021;22:244. 34429148 10.1186/s13059-021-02466-0 PMC8386078

[19] Matys V. TRANSFAC(R) and its module TRANSCompel(R): transcriptional gene regulation in eukaryotes. Nucleic Acids Res. 2006;34:D108-110. 16381825 10.1093/nar/gkj143 PMC1347505

[20] Heinz S, Benner C, Spann N, Bertolino E, Lin YC, Laslo P, et al. Simple combinations of lineage-determining transcription factors prime cis-regulatory elements required for macrophage and B cell identities. Mol Cell. 2010;38:576–89. 20513432 10.1016/j.molcel.2010.05.004 PMC2898526

[21] McLean CY, Bristor D, Hiller M, Clarke SL, Schaar BT, Lowe CB, et al. GREAT improves functional interpretation of cis-regulatory regions. Nat Biotechnol. 2010;28:495–501. 20436461 10.1038/nbt.1630 PMC4840234

[22] Kuleshov MV, Jones MR, Rouillard AD, Fernandez NF, Duan Q, Wang Z, et al. Enrichr: a comprehensive gene set enrichment analysis web server 2016 update. Nucleic Acids Res. 2016;44:W90-97. 27141961 10.1093/nar/gkw377 PMC4987924

[23] Qi Q, Cheng L, Tang X, He Y, Li Y, Yee T, et al. Dynamic CTCF binding directly mediates interactions among cis -regulatory elements essential for hematopoiesis. Blood. 2021;137:1327–39. 33512425 10.1182/blood.2020005780 PMC7955410

[24] Vermunt MW, Luan J, Zhang Z, Thrasher AJ, Huang A, Saari MS, et al. Gene silencing dynamics are modulated by transiently active regulatory elements. Mol Cell. 2023;83:715-730.e6. 36868189 10.1016/j.molcel.2023.02.006 PMC10719944

[25] Arzate-Mejía RG, Recillas-Targa F, Corces VG. Developing in 3D: the role of CTCF in cell differentiation. Development. 2018;145:dev137729. 29567640 10.1242/dev.137729 PMC5897592

[26] Yesbolatova A, Saito Y, Kitamoto N, Makino-Itou H, Ajima R, Nakano R, et al. The auxin-inducible degron 2 technology provides sharp degradation control in yeast, mammalian cells, and mice. Nat Commun. 2020;11:5701. 33177522 10.1038/s41467-020-19532-z PMC7659001

[27] Nora EP, Goloborodko A, Valton AL, Gibcus JH, Uebersohn A, Abdennur N, et al. Targeted degradation of CTCF decouples local insulation of chromosome domains from genomic compartmentalization. Cell. 2017;169:930–44. 28525758 10.1016/j.cell.2017.05.004 PMC5538188

[28] Luan J, Xiang G, Gómez-García PA, Tome JM, Zhang Z, Vermunt MW, et al. Distinct properties and functions of CTCF revealed by a rapidly inducible degron system. Cell Rep. 2021;34:108783. 33626344 10.1016/j.celrep.2021.108783 PMC7999233

[29] Kubo N, Ishii H, Xiong X, Bianco S, Meitinger F, Hu R, et al. Promoter-proximal CTCF binding promotes distal enhancer-dependent gene activation. Nat Struct Mol Biol. 2021;28:152–61. 33398174 10.1038/s41594-020-00539-5 PMC7913465

[30] Wu H-J, Landshammer A, Stamenova EK, Bolondi A, Kretzmer H, Meissner A, et al. Topological isolation of developmental regulators in mammalian genomes. Nat Commun. 2021;12:4897. 34385432 10.1038/s41467-021-24951-7 PMC8361032

[31] Huang H, Zhu Q, Jussila A, Han Y, Bintu B, Kern C, et al. CTCF mediates dosage- and sequence-context-dependent transcriptional insulation by forming local chromatin domains. Nat Genet. 2021;53:1064–74. 34002095 10.1038/s41588-021-00863-6 PMC8853952

[32] Moriguchi T, Yu L, Takai J, Hayashi M, Satoh H, Suzuki M, et al. The human GATA1 gene retains a 5’ insulator that maintains chromosomal architecture and GATA1 expression levels in splenic erythroblasts. Mol Cell Biol. 2015;35:1825–37. 25755285 10.1128/MCB.00011-15 PMC4405652

[33] Grass JA, Boyer ME, Pal S, Wu J, Weiss MJ, Bresnick EH. GATA-1-dependent transcriptional repression of GATA-2 via disruption of positive autoregulation and domain-wide chromatin remodeling. Proc Natl Acad Sci U S A. 2003;100:8811–6. 12857954 10.1073/pnas.1432147100 PMC166395

[34] Jing H, Vakoc CR, Ying L, Mandat S, Wang H, Zheng X, et al. Exchange of GATA factors mediates transitions in looped chromatin organization at a developmentally regulated gene locus. Mol Cell. 2008;29:232–42. 18243117 10.1016/j.molcel.2007.11.020 PMC2254447

[35] Lutz M, Burke LJ, Barreto G, Goeman F, Greb H, Arnold R, et al. Transcriptional repression by the insulator protein CTCF involves histone deacetylases. Nucleic Acids Res. 2000;28:1707–13. 10734189 10.1093/nar/28.8.1707 PMC102824

[36] Bresnick EH, Johnson KD. Blood disease-causing and -suppressing transcriptional enhancers: general principles and GATA2 mechanisms. Blood Adv. 2019;3:2045–56. 31289032 10.1182/bloodadvances.2019000378 PMC6616255

[37] Bresnick EH, Lee H-Y, Fujiwara T, Johnson KD, Keles S. GATA switches as developmental drivers. J Biol Chem. 2010;285:31087–93. 20670937 10.1074/jbc.R110.159079 PMC2951181

[38] Brinkman EK, Kousholt AN, Harmsen T, Leemans C, Chen T, Jonkers J, et al. Easy quantification of template-directed CRISPR/Cas9 editing. Nucleic Acids Res. 2018;46:e58. 29538768 10.1093/nar/gky164 PMC6007333

[39] Pinto EM, Chen X, Easton J, Finkelstein D, Liu Z, Pounds S, et al. Genomic landscape of paediatric adrenocortical tumours. Nat Commun. 2015;6:6302. 25743702 10.1038/ncomms7302 PMC4352712

[40] Anders S, Pyl PT, Huber W. HTSeq-A Python framework to work with high-throughput sequencing data. Bioinformatics. 2015;31:166–9. 25260700 10.1093/bioinformatics/btu638 PMC4287950

[41] Corces MR, Trevino AE, Hamilton EG, Greenside PG, Sinnott-Armstrong NA, Vesuna S, et al. An improved ATAC-seq protocol reduces background and enables interrogation of frozen tissues. Nat Methods. 2017;14:959–62. 28846090 10.1038/nmeth.4396 PMC5623106

[42] Landt SG, Marinov GK, Kundaje A, Kheradpour P, Pauli F, Batzoglou S, et al. ChIP-seq guidelines and practices of the ENCODE and modENCODE consortia. Genome Res. 2012;22:1813–31. 22955991 10.1101/gr.136184.111 PMC3431496

[43] Rao SSP, Huntley MH, Durand NC, Stamenova EK, Bochkov ID, Robinson JT, et al. A 3D map of the human genome at kilobase resolution reveals principles of chromatin looping. Cell. 2014;159(7):1665–80. 25497547 10.1016/j.cell.2014.11.021 PMC5635824

[44] Yang X, Cheng L, Xin Y, Xu J, Zhang J, Feng R, Hyle J, Qi W, Rosikiewicz W, Xu B, Li C, Xu P. CTCF is selectively required for maintaining the chromatin accessibility and gene expression in human erythropoiesis. GSE201822. 2024. https://www.ncbi.nlm.nih.gov/geo/query/acc.cgi?acc=GSE201822. 10.1186/s13059-025-03510-z PMC11869676 40022213

[45] Hyle J, Zhang Y, Wright S, Li C. Acute depletion of CTCF directly affects MYC regulation through loss of enhancer–promoter looping. GSE174528. 2019. https://www.ncbi.nlm.nih.gov/geo/query/acc.cgi?acc=GSE174528. 10.1093/nar/gkz462 PMC6648894 31127282

[46] Xu B, Wright S, Hyle J, Zhang Y, Shao Y, Fan Y, Lu R, Li C. Acute depletion of CTCF rewires genome-wide chromatin accessibility. GSE153237. 2021. https://www.ncbi.nlm.nih.gov/geo/query/acc.cgi?acc=GSE153237. 10.1186/s13059-021-02466-0 PMC8386078 34429148

[47] Qi Q, Cheng L, Tang X, He Y et al. Dynamic CTCF occupancy during differentiation rewires cis-regulatory module interactions essential for development. GSE131055. 2021. https://www.ncbi.nlm.nih.gov/geo/query/acc.cgi?acc=GSE131055.

[48] Xu P, Xu B, Tang X. FBXO11-Mediated Proteolysis of BAHD1 Relieves PRC2-dependent Transcriptional Repression in Erythropoiesis. GSE115357. 2020. https://www.ncbi.nlm.nih.gov/geo/query/acc.cgi?acc=GSE115357. 10.1182/blood.2020007809 PMC7820877 33156908

[49] Himadewi P, Wang XQD, Feng F, Gore H, Liu Y, Yu L, et al. 3′HS1 CTCF binding site in human β-globin locus regulates fetal hemoglobin expression. Elife. 2021;10:e70557. 34585664 10.7554/eLife.70557 PMC8500713

[50] Zhang X. WT-HUDEP2 cell HiC. GSM4873113. 2020. https://www.ncbi.nlm.nih.gov/geo/query/acc.cgi?acc=GSM4873113.

[51] Xu B, M.N.D, Li C, Williams J. St Jude Center for Applied Bioinformatics General Pipelines. figshare. Collection. 2022. 10.6084/m9.figshare.c.6186670.

[52] Xu B. RNA-seq. figshare. Software. 2023. 10.6084/m9.figshare.24803238.v1.

[53] Wright S, Zhao X, Rosikiewicz W, Mryncza S, Hyle J, Qi W, et al. Systematic characterization of the HOXA9 downstream targets in MLL-r leukemia by noncoding CRISPR screens. Nat Commun. 2023;14:7464. https://www.ncbi.nlm.nih.gov/geo/query/acc.cgi?acc=GSM6656049. GSM6656049. 38016946 10.1038/s41467-023-43264-5 PMC10684515

[54] Xu B. ATAC-seq QC and peak calling. figshare. Software. 2024. 10.6084/m9.figshare.13046432.v1.

[55] Xu B, M.N.D. ChIPseq and RNAseq. figshare. Software. 2022. 10.6084/m9.figshare.21045889.v1.

[56] Xu B. Hi-C and HiChIP. figshare. Software. 2022. 10.6084/m9.figshare.21002533.v1.

