# Supplementary material for: CTCF is selectively required for maintaining chromatin accessibility and gene expression in human erythropoiesis
Source: Genome Biol. 2025 Feb 28;26:44. doi: 10.1186/s13059-025-03510-z (PMC11869676; doi:10.1186/s13059-025-03510-z)
Supplement: Supplementary file 1 — Additional file 1: Supplementary Figures S1 – S7 and figure legend. [file 13059_2025_3510_MOESM1_ESM.docx]

**Supplemental Figures and Figure Legends**

**
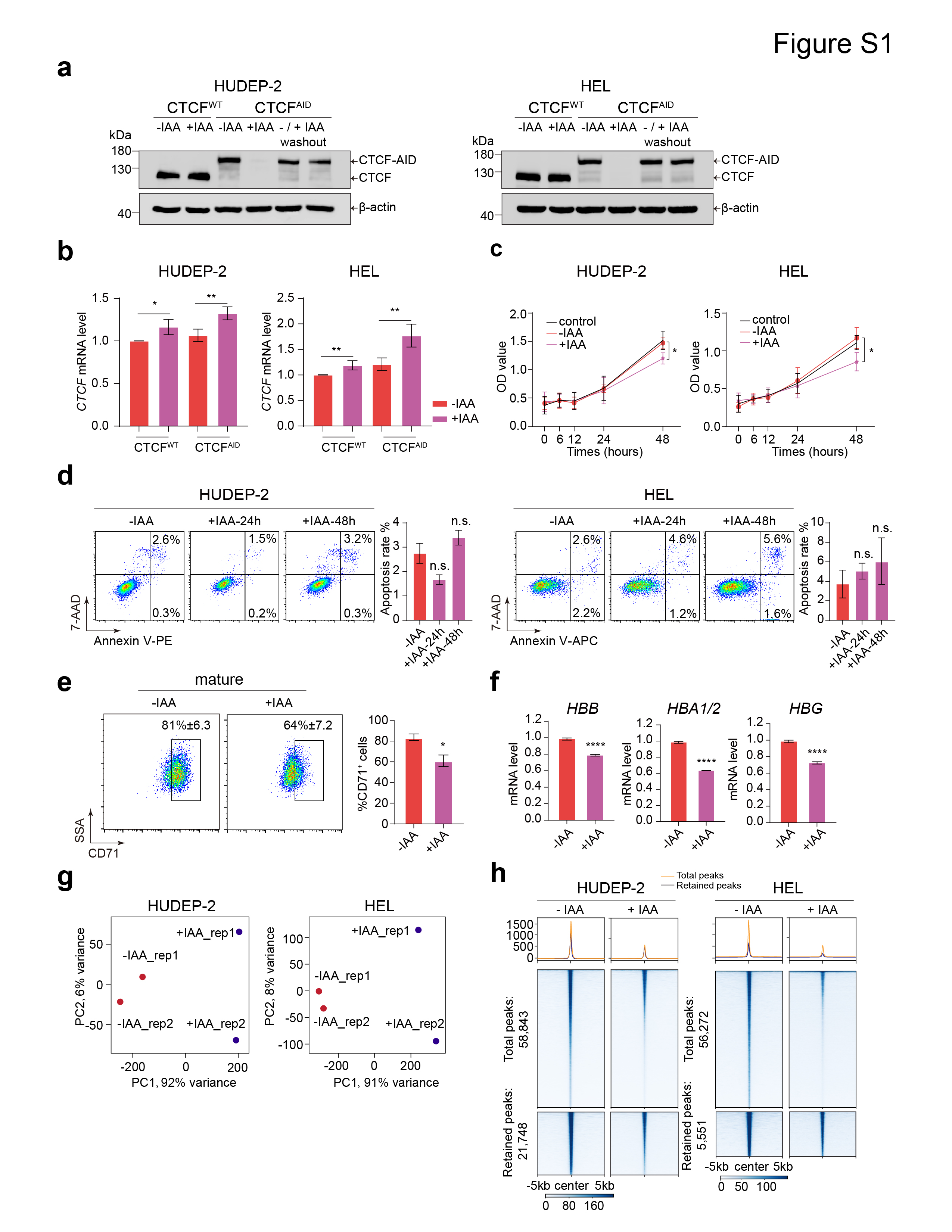
**

**Figure S1. CTCF is indispensable for the proliferation and maturation of erythroid progenitor cells.**

**(a).** Western plots showing CTCF expression in HUDEP-2 and HEL cells, including both parental wild-type and CTCF-AID clones without and with IAA treatment for 24 hours and washout of IAA for additional 24 hours. β-actin was used as a loading control.

(**b).** CTCF mRNA levels were determined in HUDEP-2 and HEL cell lines, including parental wild-type and CTCF-AID clones, without and with IAA treatment. The y-axis shows the mRNA level relative to that of β-actin. **P* < 0.05, ***P* < 0.01, ****P* < 0.001, unpaired t-test.

**(c).** The proliferation of HUDEP-2 and HEL cells after IAA treatment for 6, 12, 24, and 48 hours (highlighted in purple) was evaluated via a CCK-8 assay. The red curve represents cells treated with DMSO as the experimental control for 6, 12, 24, or 48 hours. The blank curve represents parental wild-type control cells with a normal expansion medium. **P* < 0.05, unpaired t-test.

**(d).** Flow cytometry analysis showing the apoptosis faction by the combined 7-AAD/Annexin V^+^ cells, including early and late apoptotic events without and with IAA treatment for 24 and 48 hours. The column on the right shows the quantified percentage of apoptotic cells.

**(e).** Flow cytometry plots showing CD71 expression in HEL cells on day 2 after induction of erythroid maturation without and with IAA treatment for 24 hours. The graph on the right shows the quantified percentage of CD71^+^ cells. The data are presented as the means ± SEMs of three replicates. **P* < 0.05, unpaired *t*-test.

**(f).** The *HBB, HBA1/2,* and *HBG* mRNA levels in HEL cell line erythroid maturation on day 3 without and with IAA treatment. The y-axis shows the mRNA level relative to β-actin. **** *P*< 0.0001, unpaired *t*-test.

**(g).** Principal component analysis (PCA) of CTCF ChIP-seq data from CTCF-AID HUDEP-2 and HEL cells without and with IAA treatment for 24 hours. Each condition had two independent replicates.

**(h).** Genomic heatmap centered at peak summits for the reproducible total CTCF peaks without IAA treatment and the retained peaks with IAA treatment from both HUDEP-2 and HEL cells. The CTCF binding signals were merged from two independent replicates and were shown for two conditions with or without IAA in two cell lines.


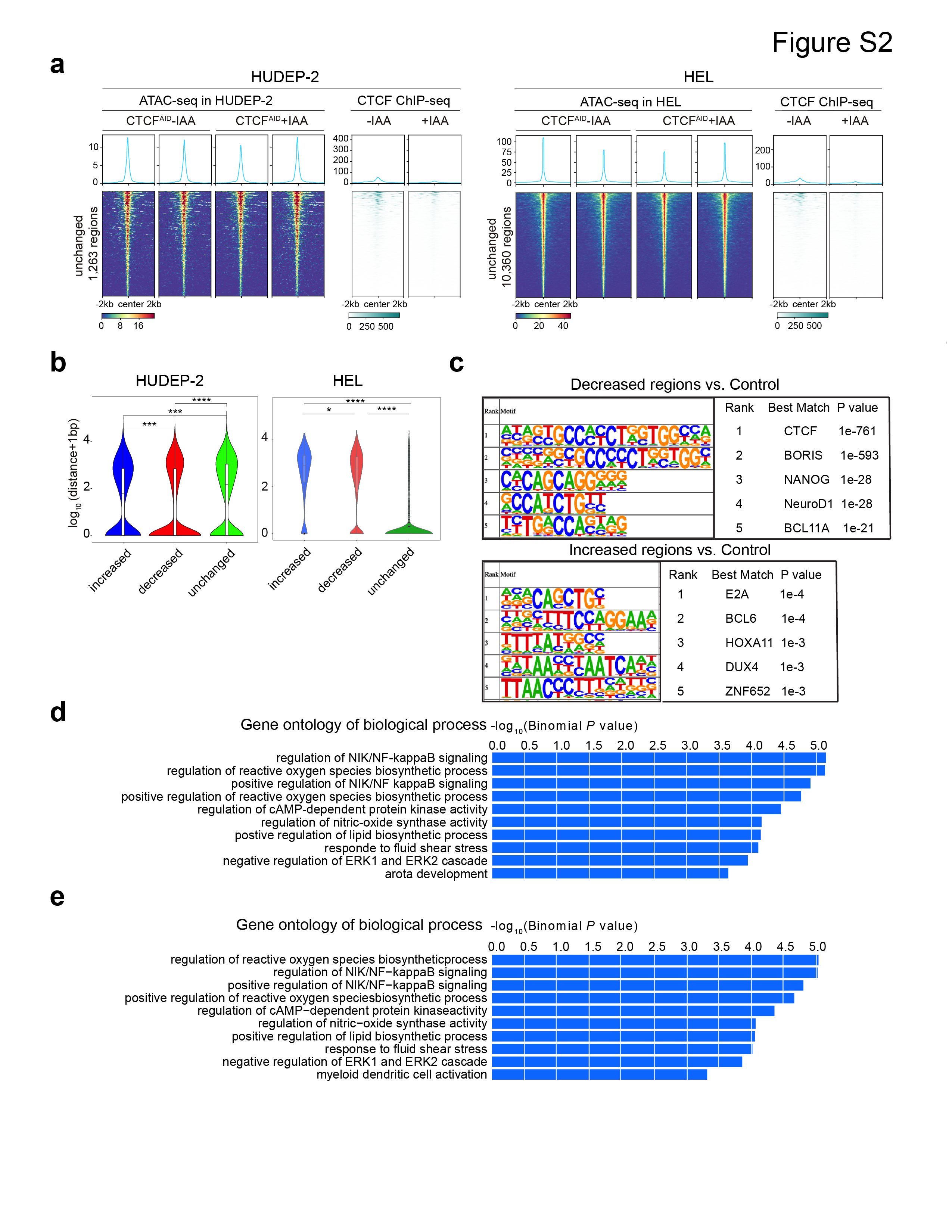


**Figure S2. CTCF depletion leads to substantial loss of chromatin accessibility in erythroid progenitor cells.**

**(a)**. Heatmap centered at the ATAC-seq nucleosome-free peaks together with the corresponding CTCF binding signal obtained via ChIP-seq for unchanged regions as controls in the HUDEP-2 (left panel) and HEL (right panel) cell lines. Unchanged regions were called based on the cutoffs of a false discovery rate (FDR) > 0.05 and a less than 2-fold change.

**(b).** Boxplots of ATAC-seq peak intensities (fragments per kilobase of peaks per million reads mapped, FPKM) in the control, decreased, and increased accessibility regions in the HUDEP-2 (left panel) and HEL (right panel) cell lines, which were used to calculate the relative distance to the annotated CTCF motifs.

**(c).** De novo motif analysis (Homer v4.9.1) for decreased regions (left panel) and increased regions (right panel) relative to unchanged regions in HUDEP-2 cells.

**(d).** Gene ontology analysis of the enriched biological processes from the decreased region from ATAC-seq data in HUDEP-2 cells. The relative *P* value was calculated via the enrichR database.

**(e).** Gene ontology analysis of the enriched biological processes from the decreased region from HEL ATAC-seq data. The relative *P* value was calculated via the enrichR database.


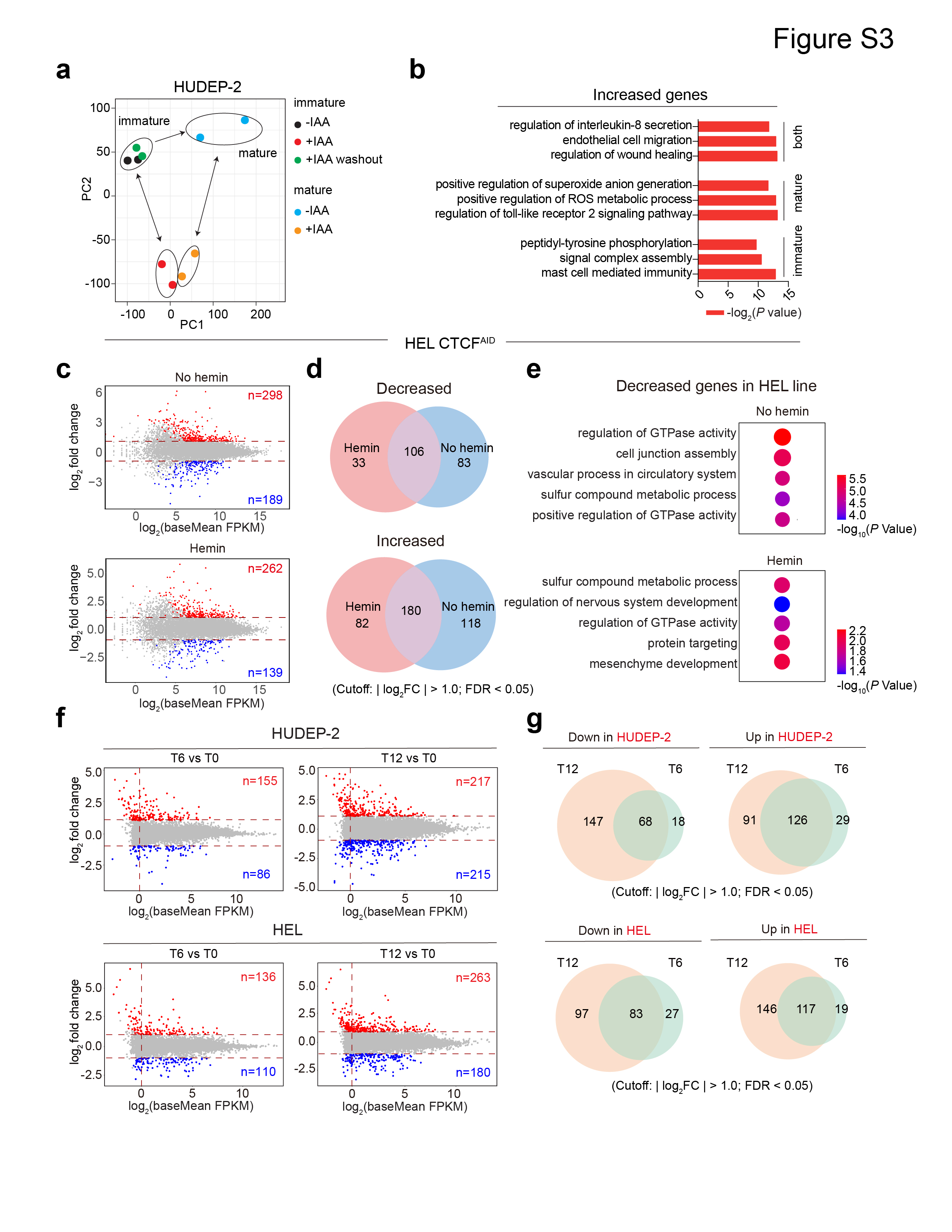


**Figure S3. CTCF is required for target gene expression in a stage-dependent manner.**

**(a).** Principal component analysis (PCA) of the transcriptomes of two individual CTCF-AID clones of HUDEP-2 without IAA treatment, with IAA treatment for 24 hours and washout of IAA for 24 hours in expansion medium, together with the corresponding groups after induction of maturation for two days. Each condition had two clone replicates.

**(b)**. Gene ontology analysis of the enriched biological processes from the increased genes in the mature, immature, and both states of HUDEP-2 cells. The relative *P* value was calculated via enrichR online software.

**(c)**. Volcano plot showing transcriptome changes in CTCF-AID clones of HEL with and without IAA treatment in the no-hemin expansion state (left panel). Volcano plot showing transcriptome changes in the CTCF-AID clones of HEL on day 3 after hemin-induced erythroid maturation with and without IAA treatment (right panel); the cutoff was based on an FDR < 0.05 and a 2-fold change.

**(d)**. Overlap analysis of decreased genes between no hemin and hemin treatment in the transcriptional level after CTCF depletion (upper panel) and overlap analysis of increased genes between no hemin and hemin treatment in the transcriptional level after CTCF depletion (lower panel).

**(e)**. Gene Ontology analysis of enriched biological processes associated with genes whose expression decreased in HEL cells with or without hemin treatment. The relative *P* value was calculated via enrichR online software.

**(f).** Volcano plot showing transcriptome changes in the CTCF-AID clones of HUDEP-2 (upper panel) and HEL (lower panel) with and without IAA treatment for 6 (T6) and 12 (T12) hours. The cutoff was based on an FDR < 0.05 and a 2-fold change.

**(g).** Overlap analysis of decreased and increased genes between 6 and 12 hours of IAA treatment at the transcriptional level after CTCF depletion in HUDEP-2 (upper panel) and HEL cells (lower panel).


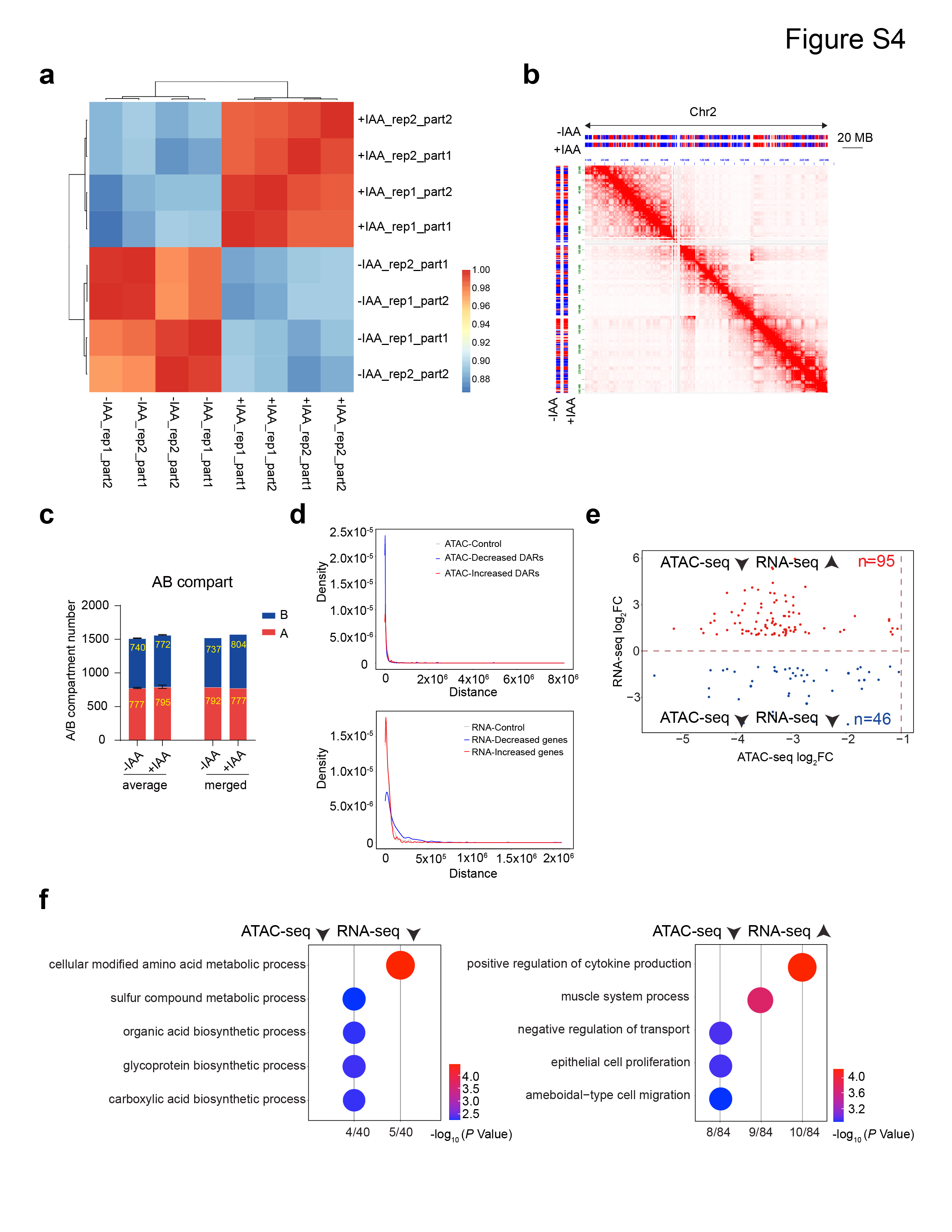


**Figure S4. Interrogate CTCF target genes based on multi-omics integrative analysis**

**(a).** Stratum-adjusted correlation coefficient at 100 kb resolution of two HEL Hi-C replicates. The values plotted were median across all chromosomes, calculated by the HiCRep R package. A higher value indicates more reproducible events.

**(b).** A representative snapshot of the chromatin compartment integrity of chromosome 2 was shown for CTCF-AID HEL cells from Hi-C data combined from two replicates without and with IAA treatment for 24 hours. Red bars indicate compartments (A); blue bars indicate compartments (B). The scale bar indicates 20 MB.

**(c).** The number of A/B compartments called from the CTCF-AID HEL is shown for cells treated with or without IAA for 24 hours. The reproducibility of the Hi-C data from two replicates was high, enabling the compilation of raw Hi-C data to recall the A/B Compartment (combined).

**(d).** The density plot shows the distances from the DARs in ATAC-seq (upper panel) and the DEGs in RNA-seq (lower panel) to the closest TAD boundaries from Hi-C in the HEL cells.

**(e).** Integration of RNA-seq and ATAC-seq analysis was performed by assessing differentially expressed genes (log_2_FC > 1, FDR < 0.05) with differential ATAC peaks (log_2_FC >1, FDR < 0.05) within 100 kb from the TSS. The upper quadrant includes genes with decreased ATAC peaks and upregulated expression (genes repressed by CTCF). The lower quadrant includes genes with decreased ATAC peaks and downregulated expression in IAA-treated cells (genes activated by CTCF).

**(f).** The top five enriched gene ontology (GO) biological process terms for the subsets of genes with decreased ATAC peaks and downregulated expression in IAA-treated cells (genes activated by CTCF) from panel (**e**) (left panel). The top five enriched gene ontology (GO) biological process terms for the subsets of genes with decreased ATAC peaks and upregulated expression in IAA-treated cells (genes activated by CTCF) from panel (**e**) (right panel).


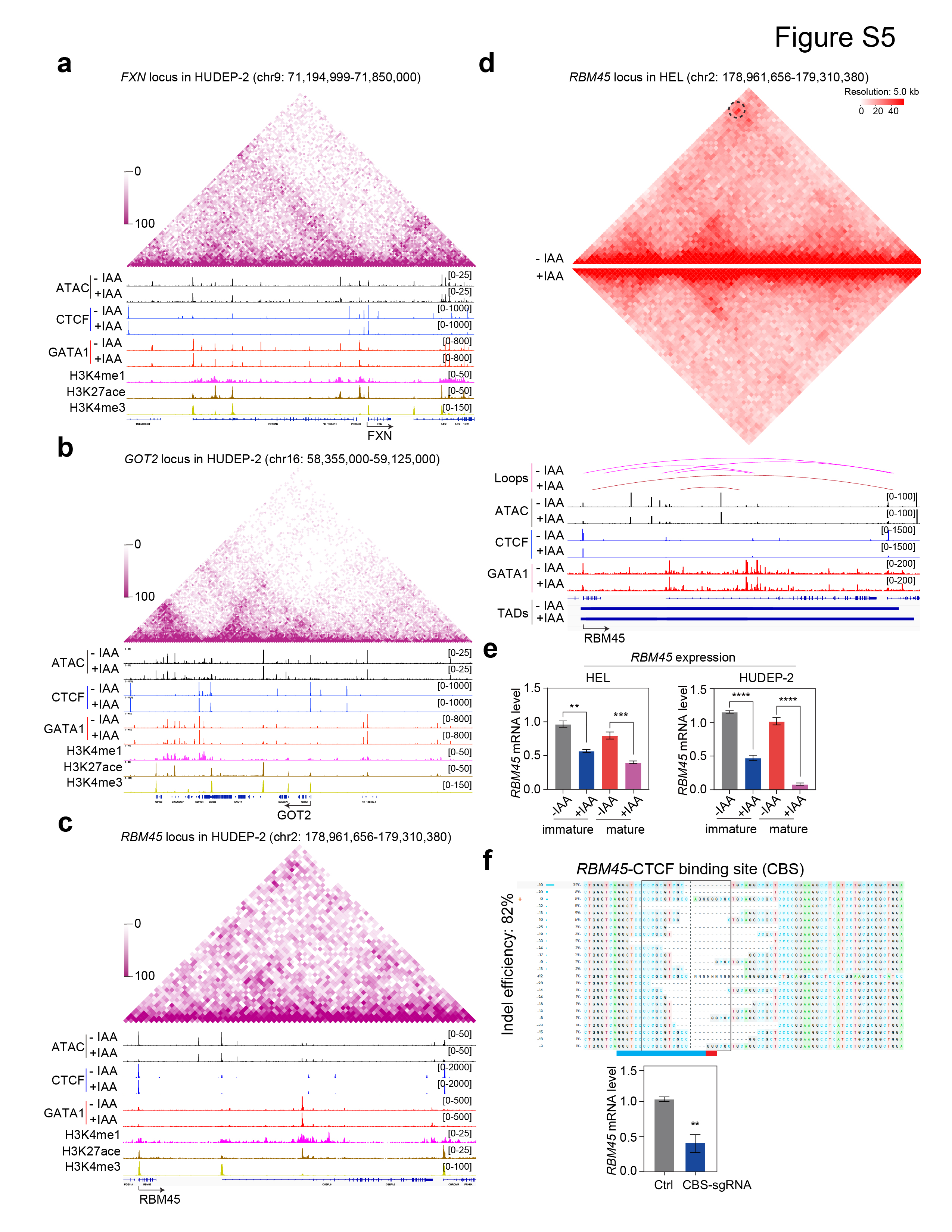


**Figure S5. Examples of the interrogate CTCF target genes based on multi-omics integrative analysis.**

**(a)-(c).** Genome-wide Hi-C interaction map and regulatory landscape around the *FXN* (**a**), *GOT2* (**b**), and *RBM45* (**c**) gene clusters in the CTCF-AID HUDEP-2 cells. ATAC-seq, CTCF ChIP-seq, GATA1 ChIP-seq, H3K4me1, H3K27ace, and H3K4me3 tracks are shown in the lower panel. “-IAA/+IAA” indicates cell cultures without or with IAA. The H3K4me3, H3K4me1, and H3K27ace ChIP-seq datasets were generated from GEO (GSE115357); the Hi-C dataset of wild-type HUDEP-2 cells was generated from GEO (GSM4873113).

**(d).** Genome-wide Hi-C interaction map and regulatory landscape around the *RBM45* gene cluster in the CTCF-AID HEL cells, with ATAC-seq, CTCF ChIP-seq, and GATA1 ChIP-seq signals together with annotated TAD domains and chromatin loops from the genome-wide Hi-C interaction map before and after IAA treatment in CTCF-AID HEL cells. The black cycle indicates the loop position identified with lost contacts after IAA treatment in CTCF-AID HEL cells.

**(e).** Plot of fragments per kilobase of peaks per million reads mapped (FPKM) values and mRNA levels relative to β-actin of *RBM45* expression in the CTCF-AID HEL cells without and with IAA treatment in both the immature and mature states. (n=3, two-tailed t-test, ***P* <0.01, ****P* <0.001).

**(f).** Measurement of *RBM45* mRNA by quantitative real-time PCR in the CBS-sgRNA-targeted bulk population relative to non-targeting sgRNA control. The expression levels were normalized to β-actin mRNA. The graph shows the results as the mean values ± SEMs for three biological replicate experiments. Two-tailed t-test, ***P* < 0.01.


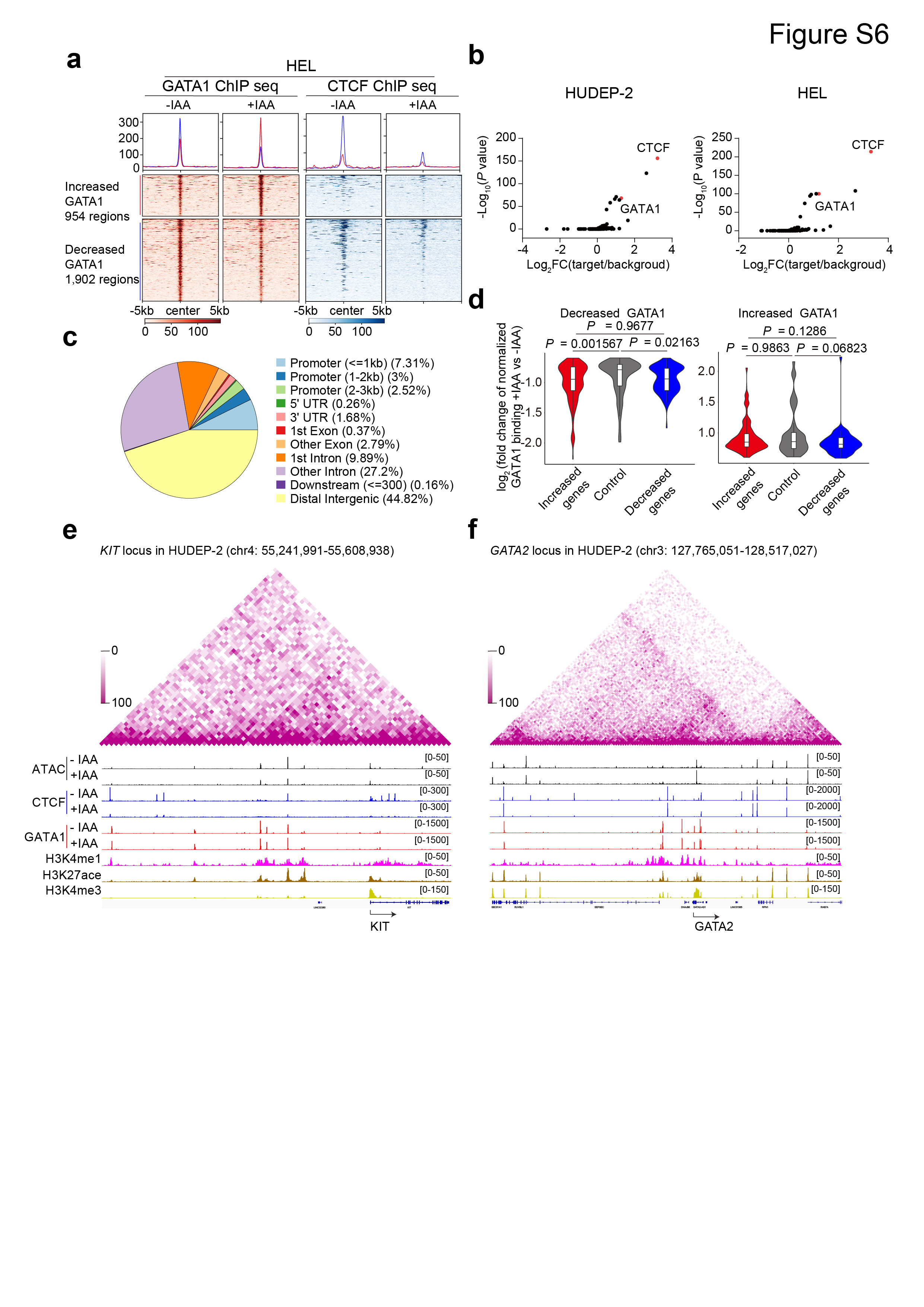


**Figure S6. CTCF is also required for a subset of GATA1-mediated gene repression**

**(a).** Genomic heatmap centered at reproducible increased GATA1 peaks and decreased GATA1 peaks from the CTCF-AID HEL cells with or without IAA treatment for 24 hours (two replicates for each condition). The right panel shows the genomic heatmap centered at the corresponding reproducible CTCF binding signal from CTCF ChIP-seq.

**(b).** Homer motif enrichment analysis for the decreased GATA1 regions after CTCF depletion revealed GATA1 and CTCF as the top enriched transcription factor motifs in both HUDEP-2 cells (left panel) and HEL cells (right panel). Enriched CTCF and GATA1 motifs are shown as red circles.

**(c).** Genomic localization of decreased GATA1 regions from ChIP-seq in HUDEP-2 cells without and with IAA treatment for 24 hours.

**(d).** Violin plot of the reduced GATA1 binding signal (fragments per kilobase of peaks per million reads mapped, FPKM) in decreased genes, increased genes, and control genes in the HUDEP-2 cells, from which the relative GATA1 binding signal was calculated. * *P* < 0.05 according to the Wilcoxon test.

**(e)-(f).** Genome-wide Hi-C interaction map and regulatory landscape around the *KIT* **(e)** and *GATA2* **(f)** gene clusters in the public dataset in the wild-type HUDEP-2 cells. ATAC-seq, CTCF ChIP-seq, GATA1 ChIP-seq, H3K4me1 ChIP-seq, H3K27ace ChIP-seq, and H3K4me3 ChIP-seq tracks are shown in the lower panel. “-IAA/+IAA” indicates cell cultures without or with IAA. The H3K4me3, H3K4me1, and H3K27ace ChIP-seq datasets were generated from GEO (GSE115357); the Hi-C dataset of wild-type HUDEP-2 cells was generated from GEO (GSM4873113).


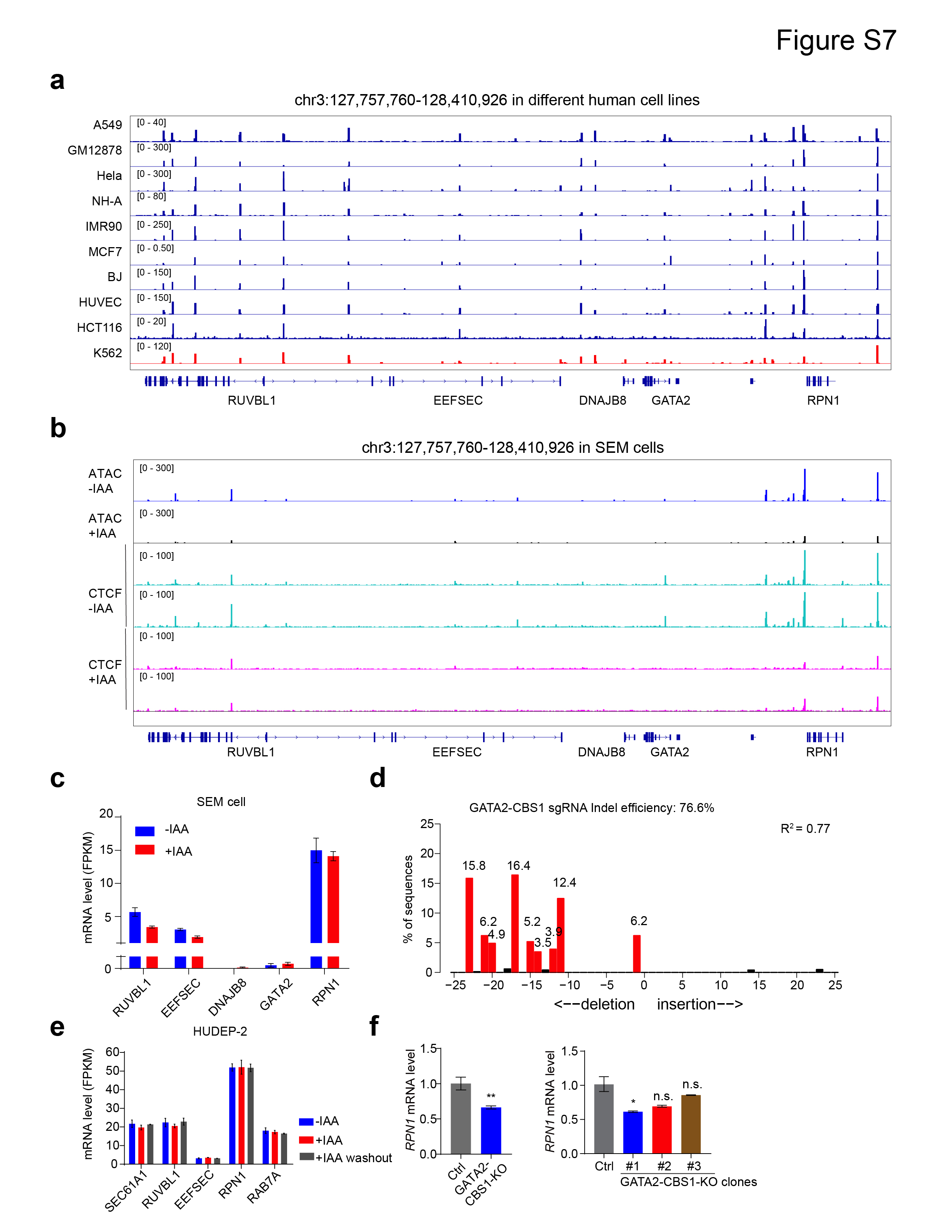


**Figure S7. CTCF represses GATA2 expression through a distal CTCF-binding site**

**(a).** ChIP-seq tracks of CTCF at the *GATA2* locus are shown from various human cancer cell lines from the ENCODE database.

**(b)**. Genome browser track analysis of the *GATA2* TAD region, including ATAC-seq signals and CTCF Cut&Run signals before and after IAA treatment in SEM cells.

**(c)**. Plot of normalized counts of *GATA2* locus genes, including *RUVBL1, EEFSEC, DNAJB8, GATA2, and RPN1*, in the CTCF-AID SEM cells without IAA treatment and with IAA treatment for 24 hours from three replicates.

**(d)**. Cas9-expressing HUDEP-2 cells were transduced with a lentiviral vector encoding sgRNA against 3’ distal GATA2-CBS1, selected with puromycin, and further collected for genomic PCR and Sanger sequencing. Indel frequency was evaluated via the TIDE-SEQ.

**(e).** Plot of normalized counts of GATA2 locus genes, including *SEC61A1, RUVBL1, EEFSEC, RPN1,* and *RAB7A*, in the CTCF-AID HUDEP-2 cells without IAA treatment, with IAA treatment for 24 hours, and with washout of IAA after 24 hours.

**(f).** Validation of the mRNA level of *RPN1* expression relative to that of β-actin in GATA2-CBS1 knockout HUDEP-2 cells at both the bulk population and cell clonal levels. (n = 3, Unpaired Student’s t-test, **P* < 0.05, ***P* < 0.01).
