# Supplementary material for: CTCF is selectively required for maintaining chromatin accessibility and gene expression in human erythropoiesis
Source: Genome Biol. 2025 Feb 28;26:44. doi: 10.1186/s13059-025-03510-z (PMC11869676; doi:10.1186/s13059-025-03510-z)

**Fig1.b**

ACTIN (the red panel) blot (HUDEP-2):


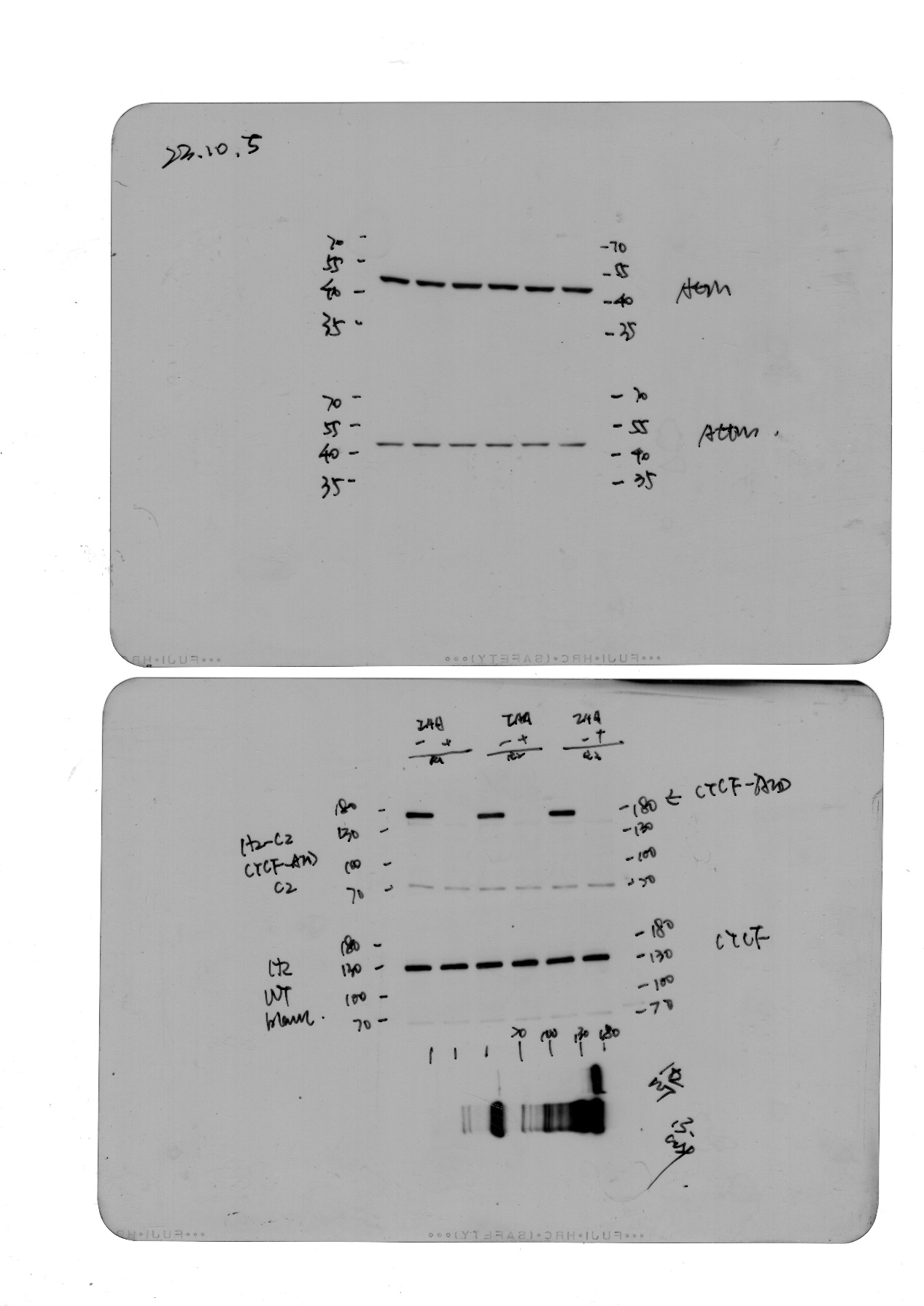


CTCF (the red panel) blot (HUDEP-2):

**
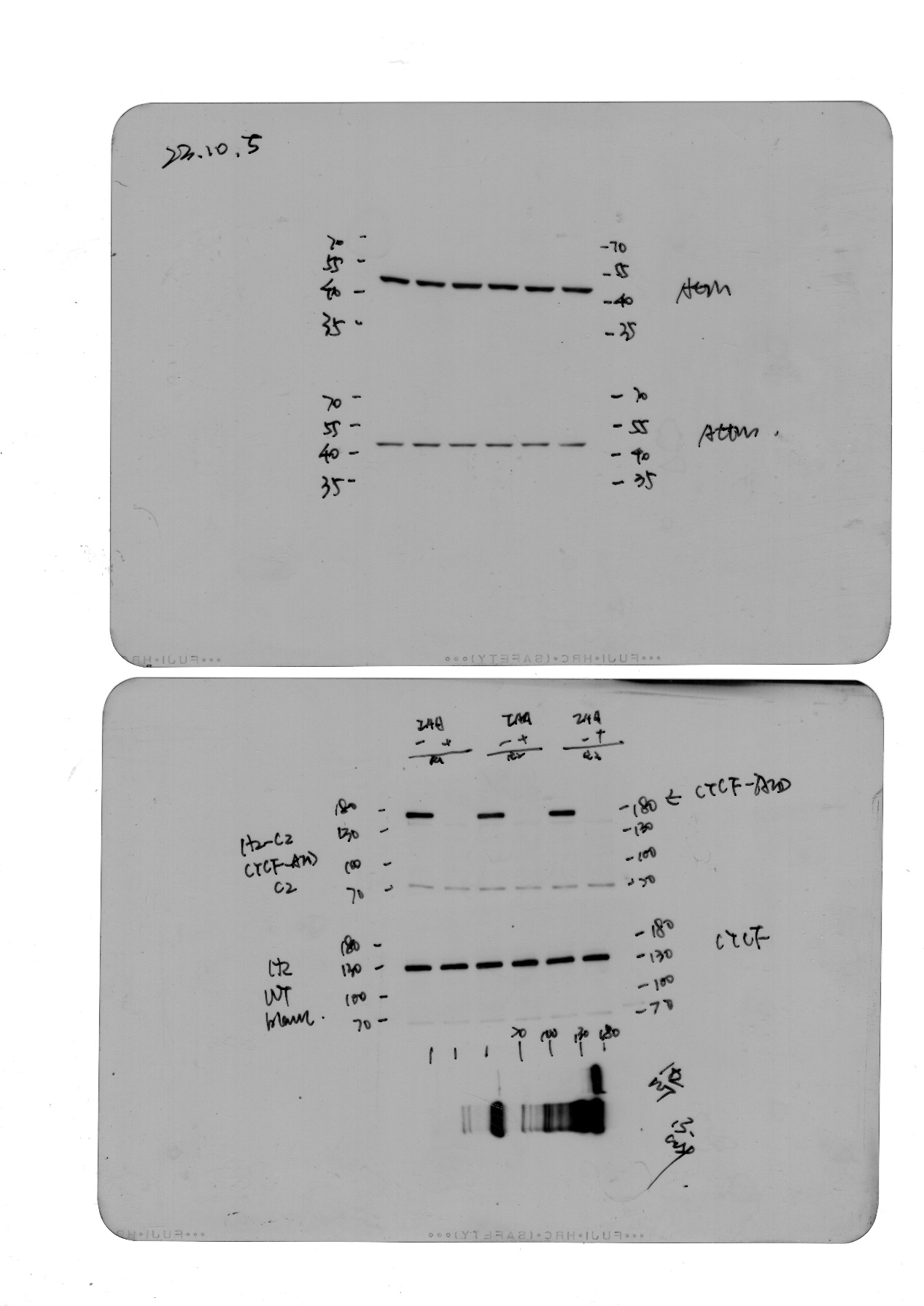
**

ACTIN (the red panel) blot (HEL):


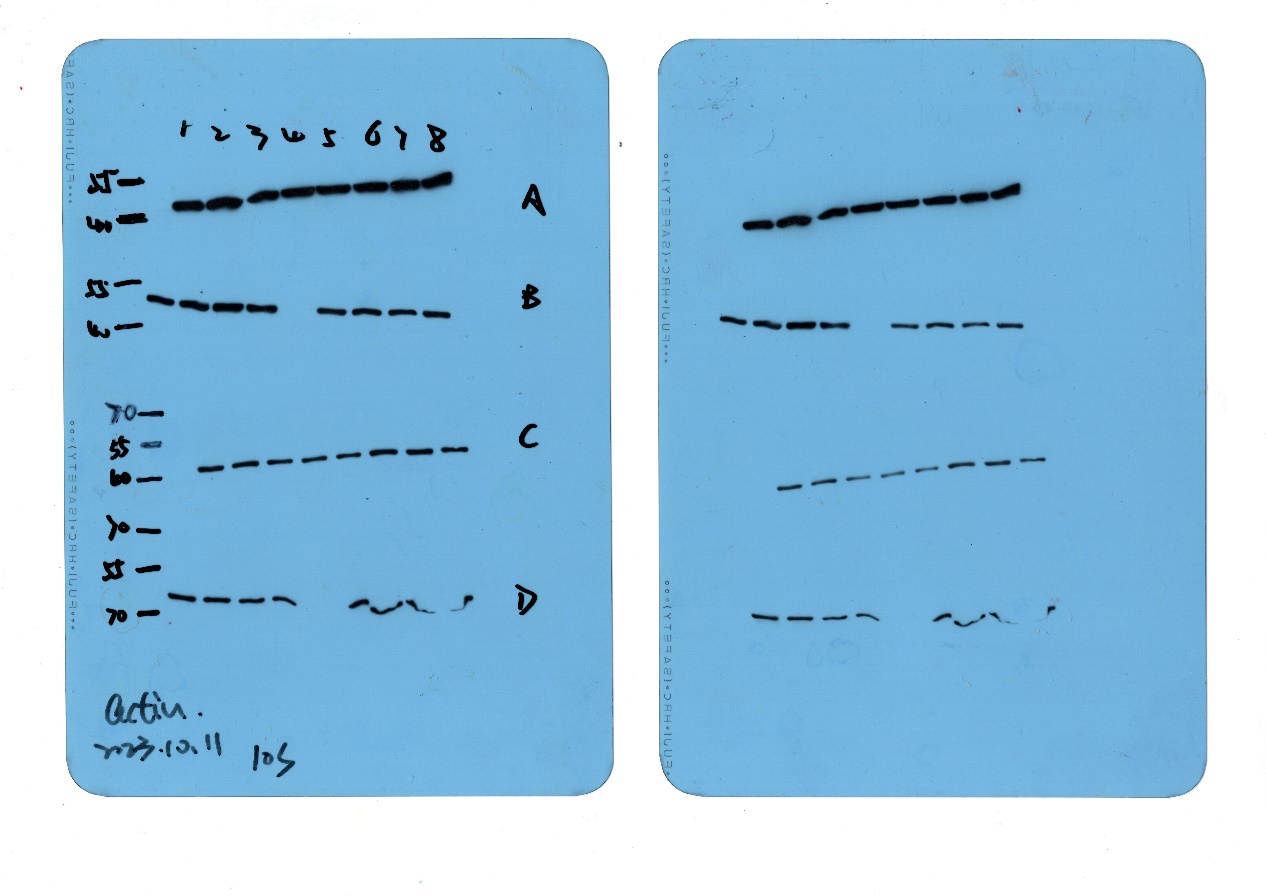


CTCF (the red panel) blot (HEL):


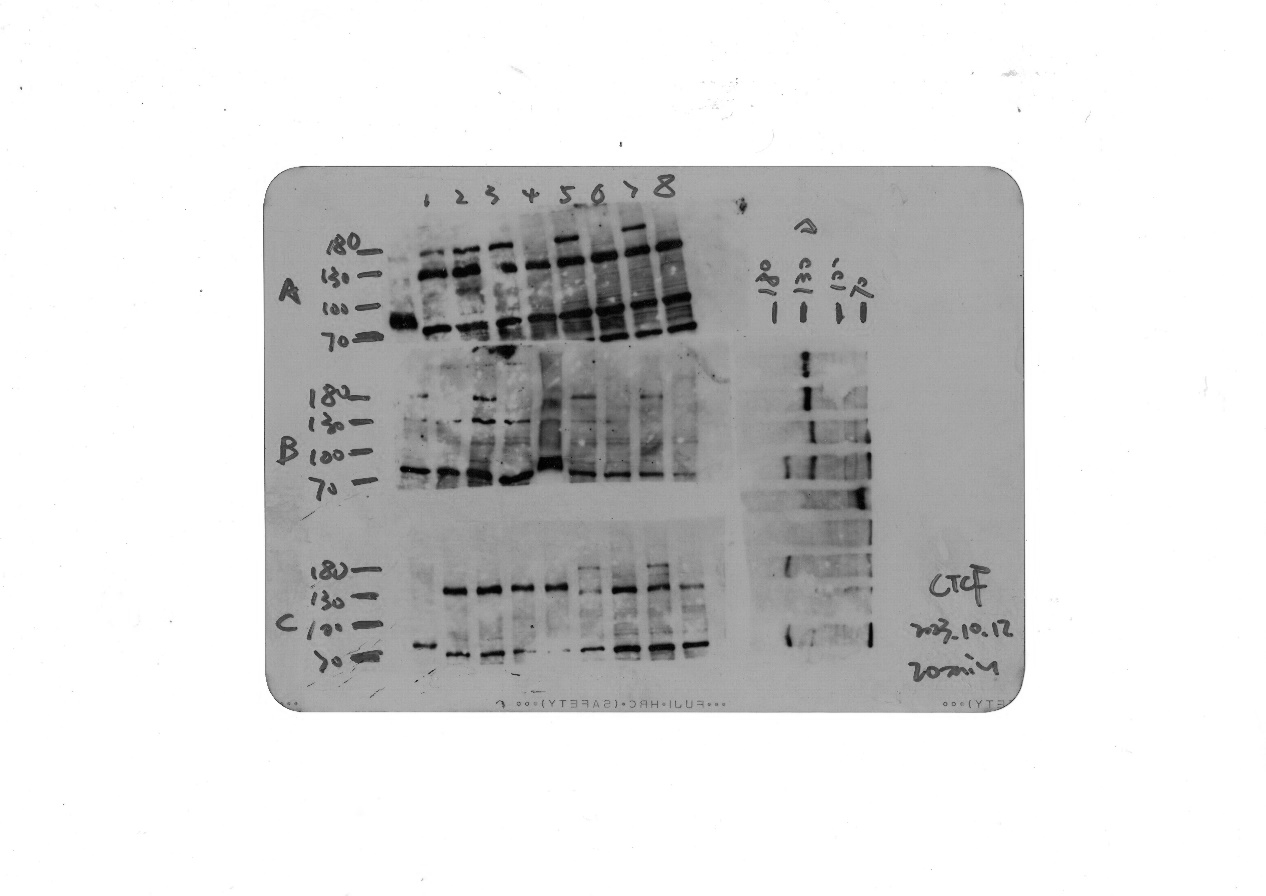


**Figure S1.a**

ACTIN blot (HUDEP-2):





CTCF blot (HUDEP-2):

**

**

ACTIN blot (HEL):





CTCF blot (HEL):





**Fig5.h**

ACTIN (the red panel) blot (HUDEP-2):


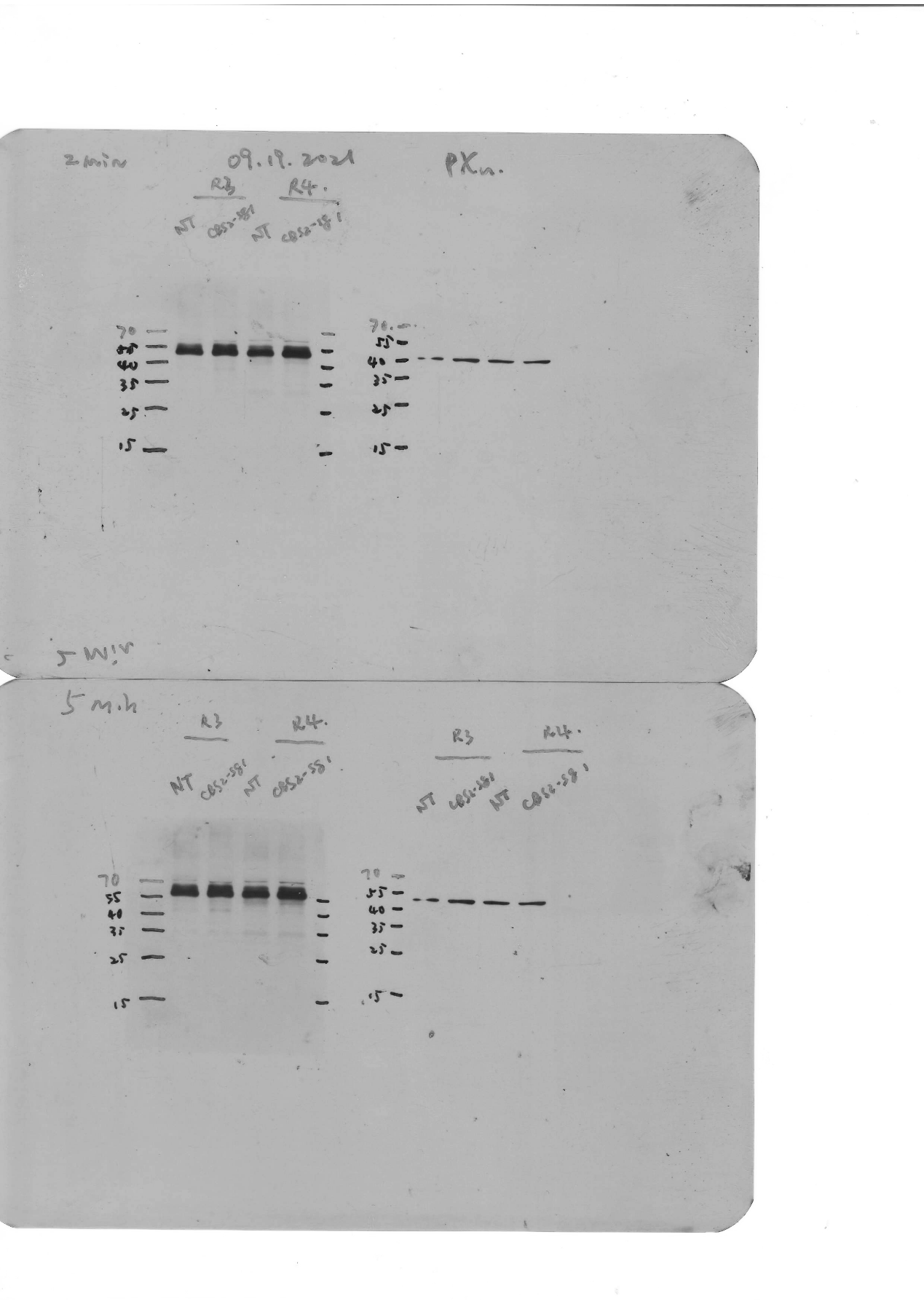


GATA2 (the red panel) blot (HUDEP-2):


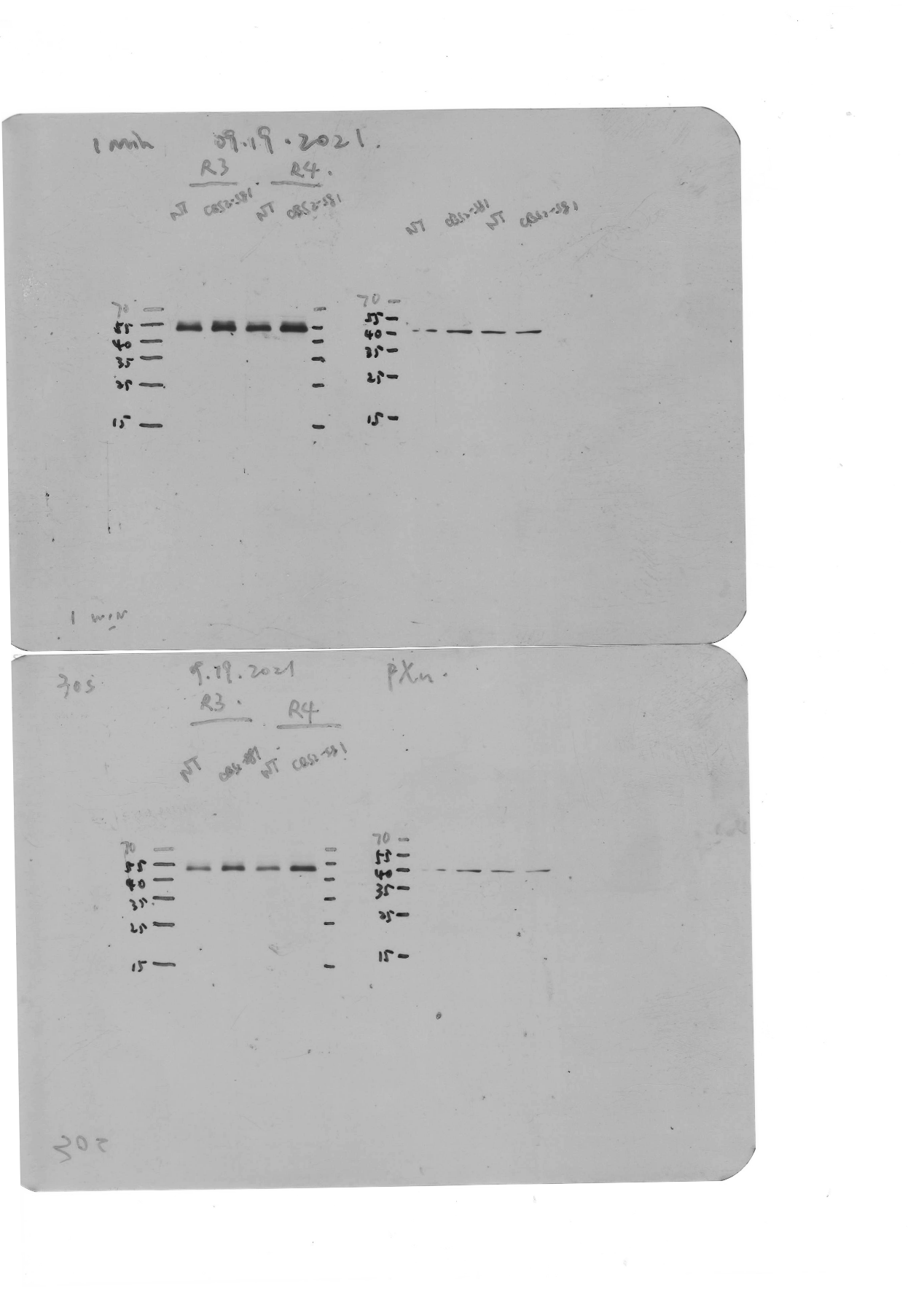

Supplement: Supplementary file 14 — Additional file 14. Uncropped images for the blots in figure 1b, figure 5h and supplementary figure S1a. [file 13059_2025_3510_MOESM14_ESM.docx]
